# Capsular Polysaccharide Production in Bacteria of the *Mycoplasma* Genus: A Huge Diversity of Pathways and Synthases for So‐Called Minimal Bacteria

**DOI:** 10.1111/mmi.15325

**Published:** 2024-10-30

**Authors:** Manon Vastel, Corinne Pau‐Roblot, Séverine Ferré, Véronique Tocqueville, Chloé Ambroset, Corinne Marois‐Créhan, Anne V. Gautier‐Bouchardon, Florence Tardy, Patrice Gaurivaud

**Affiliations:** ^1^ ANSES‐Laboratoire de Lyon, VetAgro Sup, UMR Mycoplasmoses Animales Université de Lyon Lyon France; ^2^ ANSES‐Laboratoire de Ploufragan‐Plouzané‐Niort Unité Mycoplasmologie, Bactériologie et Antibiorésistance Ploufragan France; ^3^ UMRT INRAE 1158 BioEcoAgro – Biologie des Plantes et Innovation Université de Picardie Jules Verne, UFR des Sciences Amiens France

**Keywords:** capsular polysaccharide, glucofuranose, glycosyltransferase, *Mycoplasma* spp., synthase

## Abstract

Mycoplasmas are wall‐less bacteria with many species spread across various animal hosts in which they can be pathogenic. Despite their reduced anabolic capacity, some mycoplasmas are known to secrete hetero‐ and homopolysaccharides, which play a role in host colonization through biofilm formation or immune evasion, for instance. This study explores how widespread the phenomenon of capsular homopolysaccharide secretion is within mycoplasmas, and investigates the diversity of both the molecules produced and the synthase‐type glycosyltransferases responsible for their production. Fourteen strains representing 14 (sub)species from four types of hosts were tested in vitro for their polysaccharide secretion using both specific (immunodetection) and nonspecific (sugar dosage) assays. We evidenced a new, atypical homopolymer of β‐(1 → 6)‐glucofuranose (named glucofuranan) in the human pathogen *Mycoplasma (M.) fermentans*, as well as a β‐(1 → 6)‐glucopyranose polymer for the turkey pathogen *M. iowae* and galactan (β‐(1 → 6)‐galactofuranose) and β‐(1 → 2)‐glucopyranose for *M. bovigenitalium* infecting ruminants. Sequence and phylogenetic analyses revealed a huge diversity of synthases from varied *Mycoplasma* species. The clustering of these membrane‐embedded glycosyltransferases into three main groups was only partially correlated to the structure of the produced homopolysaccharides.

## Graphical abstract

Synthases are membrane‐embedded glycosyltransferases enabling the polymerization and secretion of homopolysaccharides that can form a capsule around mycoplasma cells. The nature of the capsular polymer is tightly linked to different structural features of the synthase. So far, at least four different types of capsular homopolymers have been evidenced in mycoplasmas, one of which—glucofuranan (β‐(1 → 6)‐glucofuranose)—has never been described in bacteria.

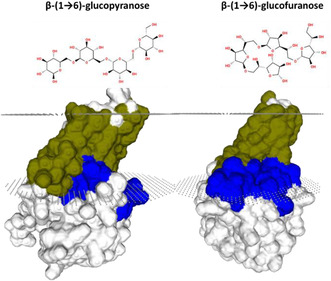

## 1 Introduction

Mycoplasmas are fast‐evolving bacteria that have derived from Gram‐positive ancestors by massive gene losses (Sirand‐Pugnet et al. 2007). The small genomes (580 to 1840 kbp) that result have notably lost the genes involved in peptidoglycan synthesis on the one hand, resulting in wall‐less cells, and in several metabolic pathways on the other, resulting in fastidious growth in vitro. However, they have retained the ability to self‐replicate in axenic media (Breuer et al. 2019). With more than 150 species, the genus *Mycoplasma* (*M*.) includes several successful pathogens among various hosts, whether human or livestock animals (Brown 2018). Polysaccharides, which may be either cell‐linked (capsular polysaccharides, CPSs) or cell‐free (exopolysaccharides, EPSs), are considered potential mycoplasmal virulence factors (Daubenspeck, Jordan, and Dybvig 2014; Gaurivaud and Tardy 2022). Most often, the secreted polysaccharides are involved in biofilm formation, such as the N‐acetylglucosamine polymer in the human atypical pneumonia agent *M. pneumoniae* (Simmons et al. 2013), or the poly N‐acetylglucosamine polysaccharide of *M. genitalium*, a mycoplasma responsible for urogenital infections in humans (Daubenspeck et al. 2020), or glucose/galactose polymers of the murine pathogen *M. pulmonis* (Daubenspeck et al. 2009). Within the host, the biofilm structure protects mycoplasmas against the action of complement, macrophage, and antimicrobial peptides (Simmons and Dybvig 2007). However, in some species, homopolysaccharides—not necessarily organized into a biofilm—have been assigned different roles depending on their structure and localization (cell‐attached or free). The galactan (β‐(1 → 6)‐galactofuranose) secreted by some species of the cluster *M. mycoides* (Bertin et al. 2015; Plackett and Buttery 1958; Plackett and Buttery 1964) is involved in serum resistance when capsular, or has an anti‐inflammatory effect when secreted as cell‐free (Gaurivaud et al. 2014; Totte et al. 2015). The β‐(1 → 6)‐glucan (β‐(1 → 6)‐glucopyranose) produced by some strains of *M. feriruminatoris*, *M. agalactiae*, and *M. mycoides* subsp. *capri* was associated with an increased serum susceptibility in *M. agalactiae* when secreted as a CPS (Ambroset et al. 2017; Gaurivaud et al. 2016). In contrast, the role of β‐(1 → 2)‐glucan (β‐(1 → 2)‐glucopyranose) produced by several strains of *M. capricolum* subsp. *capricolum*, one of the contagious agalactia bacteria found among goats, and *M. capricolum* subsp. *capripneumoniae*, the causal agent of contagious caprine pleuropneumonia, has yet to be unraveled (Bertin et al. 2015). Strains from several other species are known to produce a biofilm or a capsule, but their polysaccharide composition remains unknown (Daubenspeck, Jordan, and Dybvig 2014). This is the case for the two human pathogens *M. fermentans* (Awadh et al. 2021) and *M. penetrans* (Neyrolles et al. 1998), for *M. hyopneumoniae* involved in porcine enzootic pneumonia (Tassew et al. 2017), for bird pathogens *M. gallisepticum* (Chen et al. 2012) and *M. synoviae* (Kang et al. 2023) and for several ruminant species such as *M. bovis* (Chen et al. 2018), *M. ovipneumoniae* (Niang et al. 1998), *M. arginini* (Abdelazeem et al. 2020), and *M. dispar* (Almeida and Rosenbusch 1991).

The biosynthetic pathways behind the production and release of homopolysaccharides in *Mycoplasma* spp. are being increasingly unraveled and enzymes for uridine diphosphate (UDP) sugar synthesis and glycosyltransferases (GTs) catalyzing polymerization of the activated sugar monomer to generate homopolysaccharides have been identified and described, so far only in ruminants (Bertin et al. 2015, 2013; Gaurivaud et al. 2016; Schieck et al. 2016). Among GTs, two synthases one involved in the polymerization of UDP‐galactofuranose into of galactan homopolymer and its secretion in *M. mycoides* subsp. *mycoides* and the other in *M. agalactiae* β‐(1 → 6)‐glucan synthesis (polymerization of UDP‐glucose into β‐(1 → 6)‐glucan and secretion) have been characterized in details and further used as models to retrieve other homopolysaccharide secretion pathways in other *Mycoplasma* species such as *M. capricolum* and *M. feriruminatoris* (Ambroset et al. 2017; Bertin et al. 2015). Synthases are membrane‐embedded GTs that belong to the GT2 family and are named after one of the four pathways described for exopolysaccharide synthesis in bacteria (Schmid 2018). Synthases share a typical structure comprising a cytoplasmic loop bearing the GT active sites, surrounded by transmembrane helices (TMHs) forming a channel allowing the translocation of the polymer across the cytoplasmic membrane (Low and Howell 2018). For the other pathways, the polymerization are catalyzed by cytoplasmic GT and the secretion by transmembrane proteins complexes (Wzy/Wzx‐dependent pathway and ABC transporter‐dependent pathway) or by a single extracellular enzyme which hydrolyzes disaccharides and polymerizes one of the monosaccharide released into a homopolysaccharide (sucrase pathway) (Schmid 2018).

Identification of the transmembrane helices of synthases was used as a “signature” to differentiate homopolysaccharide synthase from other GT2 glycosyltransferases (Gaurivaud et al. 2016)—without any transmembrane domain—involved in glycolipids biosynthesis for instance, that is, GT catalyzing the addition of one or two sugar units to glycerolipids (Andres, Martinez, and Planas 2011; Klement et al. 2007). In ruminant mycoplasmas, we have previously attempted to use structural features such as the number of TMHs and the motif holding the growing glycan chain in the active site of the synthases, the so‐called “QxxRW” motif, to predict the nature of the polysaccharide produced (Gaurivaud et al. 2016).

The present work was conducted to (i) search for new CPSs/EPSs synthesized in vitro in an enlarged set of *Mycoplasma* species from different animal hosts, (ii) predict or determine their chemical structure, and (iii) analyze in silico the corresponding biosynthesis pathway with a focus on the sequence‐function relationships of synthases. We were aiming to define how universal homopolysaccharide synthesis is within the *Mycoplasma* genus, and to shed light on the diversity of the different synthases involved in their synthesis and release.

## 2 Results

### 2.1 Polysaccharide Secretions In Vitro

Fourteen *Mycoplasma* species distributed over four major host groups (mammals and birds) were selected for polysaccharide secretion screening in vitro (Table 1). We excluded species for which data were already available, such as *M. genitalium* (Daubenspeck et al. 2020), *M. pneumoniae* (Simmons et al. 2013), *M. mycoides* (Bertin et al. 2013), *M. capricolum* (Bertin et al. 2015), *M. agalactiae* (Gaurivaud et al. 2016), and *M. feriruminatoris* (Ambroset et al. 2017), and focused on species of medical/veterinary importance or species with uncertain contribution to clinical signs but for which a synthase homolog was predicted in silico, such as *M. moatsii*, *M. bovigenitalium*, and *M. iowae* (Tables 1 and S1). Glycosyltransferases from *Mycoplasma* spp. were retrieved from the CAZy database. Among these GTs those with predicted multiple TMHs were considered as potential synthases. All predicted synthases belong to the GT2 family.

**TABLE 1:** In vitro detection and quantification of capsular polysaccharides (CPSs) and exopolysaccharides (EPSs) in selected *Mycoplasma* (*M.*) strains.

Host	Species/subspecies (alternative genus name^a^)	Strain	CFUs or CCUs/mL^b^		CPS μg/mL^c^	EPS μg/mL^c^	CPS pathway^d^	Colony blotting^e^		
			T0	T72				Galactan	β‐(1 → 2)‐glucan	β‐(1 → 6)‐glucan
Ruminant	*M. mycoides* subsp. *capri* (*Mycoplasma*)	95010	5.8 × 10^9^ ± 8.210^7^	5.2 × 10^8^ ± 2.7 × 10^7^	2.7 ± 0.2	n.d.	SP	+	−	−
	*M. ovipneumoniae* ^f^ (*Mesomycoplasma*)	L14811	3.2 × 10^7^ ± 6.3 × 10^6^	< 10	n.d.	n.d.	U	ND	ND	ND
	*M. bovis* (*Mycoplasmopsis*)	L11436	4.0 × 10^9^ ± 2.7 × 10^8^	9.4 × 10^4^ ± 3.6 × 10^3^	n.d.	n.d.	SP	−	−	+
	*M. bovirhinis* (*Mycoplasmopsis*)	L11513	7.6 × 10^8^ ± 1.1 × 10^7^	8.1 × 10^5^ ± 2.0 × 10^3^	n.d.	n.d.	U	ND	ND	ND
	*M. arginini* (*Mycoplasmopsis*)	2230 (G230)	5.8 × 10^8^ ± 1.4 × 10^7^	< 10	n.d.	n.d.	U	ND	ND	ND
	*M. bovigenitalium* (*Mycoplasmopsis*)	51080	1.3 × 10^9^ ± 5.4 × 10^7^	< 10	n.d.	n.d.	SP	+	+	−
Primate	*M. fermentans* (*Mycoplasmopsis*)	PG18	6.7 × 10^8^ ± 9.5 × 10^7^	1.2 × 10^4^ ± 1.4 × 10^3^	25.0 ± 0.2	9.3 ± 0.1	SP	−	−	−
	*M. moatsii* (*Mesomycoplasma*)	NCTC10158	2.1 × 10^6^	1.2 × 10^5^ ± 1.2 × 10^4^	10.3 ± 0.1	4.9 ± 0.1	SP	−	−	−
Poultry	*M. iowae* (*Malacoplasma*)	695	1.1 × 10^8^ CCU/mL	UC	8.9 ± 0.1	n.d.	SP	−	−	+
	*M. gallisepticum* (*Mycoplasmoides*)	ATCC 15302	1.08 × 10^10^ CCU/mL	1.08 × 10^6^ CCU/mL	n.d.	n.d.	U	ND	ND	ND
	*M. synoviae* (*Mycoplasmopsis*)	WVU 1853	2.32 × 10^10^ CCU/mL	≤ 50 CCU/mL	n.d.	n.d.	U	ND	ND	ND
Swine	*M. hyopneumoniae* (*Mesomycoplasma*)	699	2 × 10^6^ CCU/mL	UC	n.d.	n.d.	U	ND	ND	ND
	*M. flocculare* (*Mesomycoplasma*)	18	UC	5 × 10^6^ CCU/mL	n.d.	n.d.	U	ND	ND	ND
	*M. hyorhinis* (*Mesomycoplasma*)	394	5 × 10^10^ CCU/mL	1.85 × 10^5^ CCU/mL	n.d.	n.d.	U	ND	ND	ND

^a^ Alternative genus names proposed by Gupta et al. (2018).

^b^ Mycoplasma concentrations were determined as CFUs (colony‐forming units)/mL or when mentioned, CCUs (color‐changing units)/mL, at the beginning (T0, 0 h) and at the end (T72, 72 h) of incubation in CMRL (see Materials and Methods). The concentration of some species could not be determined (uncountable, UC).

^c^ CPSs (capsular polysaccharides) and EPSs (exopolysaccharides) in μg/mL ± standard deviation; n.d.: not detected (or the concentration was below 2 μg/mL, see text).

^d^ SP (synthase pathway, see Table S1): prediction from the genomic data of the selected species of a synthase pathway; U (unknown): a synthase pathway was not detected from the genomic data of the species, other potential polysaccharide pathways are unknown.

^e^ Colony blotting was performed only when CPSs or EPSs were detected or when a polysaccharide synthase‐dependent pathway was predicted from genomic data. ND: not done; −, a polysaccharide was not detected by colony blotting; +, a polysaccharide was detected by colony blotting.

^f^ For *M. ovipneumoniae*, the CMRL medium was supplemented with glucose (4 g/L) in order to maximize capsule synthesis as described by Jiang et al. (2017).

CPSs and EPSs were extracted as previously described (Gaurivaud et al. 2016) and their concentration determined by a phenol‐sulfuric assay for which the limit of quantification had been previously set at 0.8 μg/mL in our experimental conditions (Gaurivaud et al. 2016). Using *M. mycoides* subsp. *capri* strain 95010 as a positive control known to secrete a galactan CPS (Bertin et al. 2015), CPS values > 2 μg/mL were considered significant. This allowed us to eliminate other glycoconjugates that could result in low signals (Bertin et al. 2015). In our experimental conditions, *M. fermentans* PG18, *M. iowae* 695 (ATCC 33552), and *M. moatsii* NCTC10158 were shown to produce huge quantities of CPSs, while only *M. fermentans* and *M. moatsii* were shown to also produce EPSs (Table 1).

**FIGURE 1:**
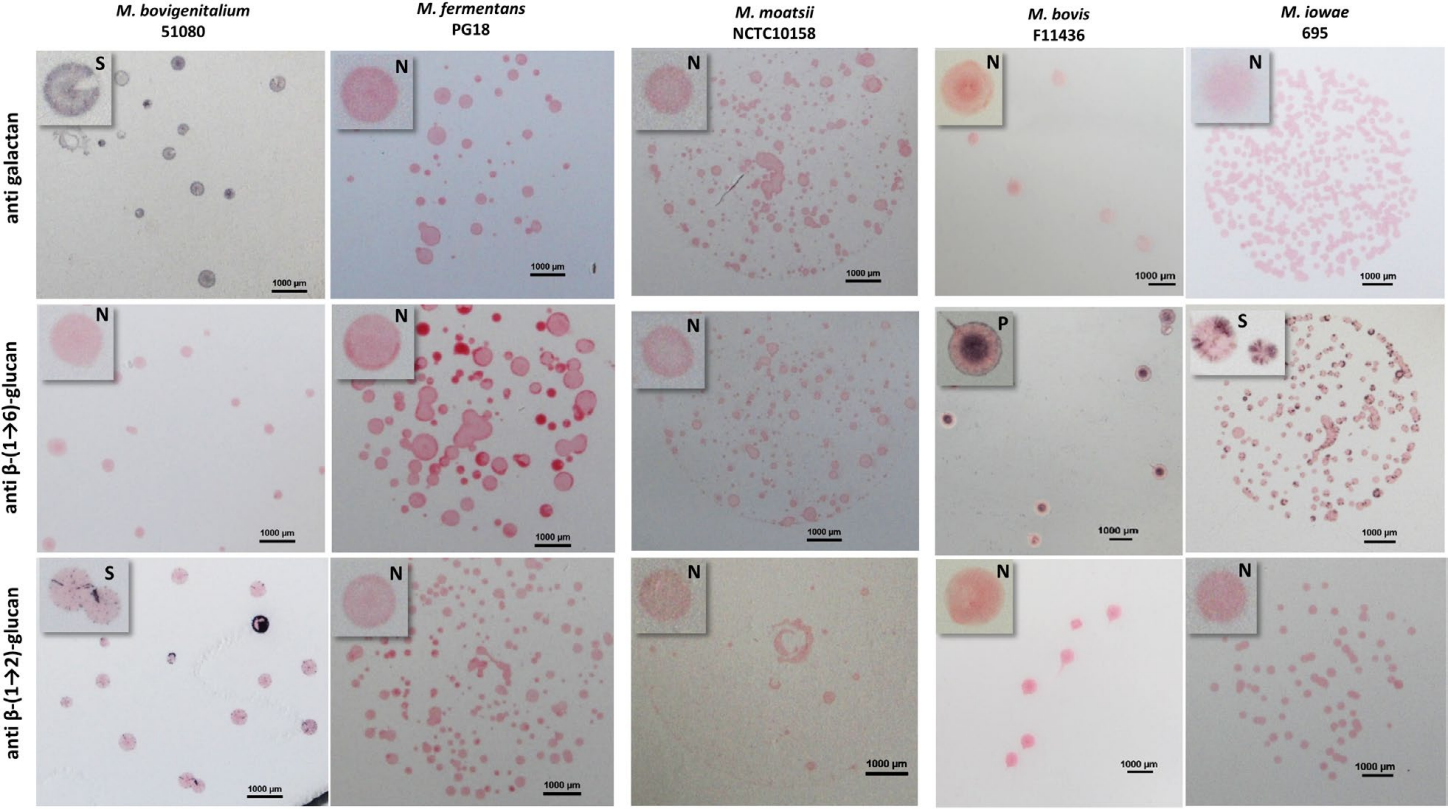
Colony immunostaining with anti galactan, anti β‐(1 → 6)‐glucan, and anti β‐(1 → 2)‐glucan specific antibodies against *M. bovigenitalium* 51080, *M. fermentans* PG18, *M. moatsii* NCTC10158, *M. bovis* F11436, and *M. iowae* 695 strains cultivated on PPLO agar plates. Colonies marked by antibodies are dark purple/grey (positive, P). Colonies not recognized by antibodies are pink due to counterstaining with Ponceau red (negative, N). “S” indicates sectored colonies. For *M. bovigenitalium* when using the anti galactan antibody, the Ponceau red counterstaining was omitted (see text); hence, part of colonies are not colored at all. Scale bar = 1 mm.

For several species (with or without a predicted synthase pathway), the absence of EPS/CPS detection could have resulted from a low viability in CMRL (Connaught Medical Research Laboratories) medium at T72 (Table 1). For species for which a putative synthase pathway has been predicted in silico (Tables 1 and S1), we further ran colony blotting assays using specific antibodies targeting the three main homopolymers produced by synthases described so far in mycoplasma, namely galactan, β‐(1 → 2)‐glucan, and β‐(1 → 6)‐glucan (Figures 1 and S1). We aimed to improve the sensitivity of detection and overcome any problem of low CMRL viability and/or antigenic variation and to potentially identify the polymer produced. *M. mycoides* subsp. *capri* strain 9510, *M. agalactiae* strain 14628 and *M. capricolum* subsp. *capricolum* F10190 previously demonstrated to produce galactan, β‐(1 → 6)‐glucan and β‐(1 → 2)‐glucan, respectively, were used as control (Figure S1). *M. iowae* 695 was shown to produce a β‐(1 → 6)‐glucan CPS—confirming our previous detection from culture broth—with some phase variations. For *M. bovis* F11436, colonies were positively stained, albeit atypically (only the center of the colony was stained), with the anti β‐(1 → 6)‐glucan although we failed to purify and detect a CPS from broth culture. *M. bovigenitalium* 51080 colonies were shown to produce galactan, although the staining was weak, as well as β‐(1 → 2)‐glucan, both expressions being under phase variation resulting in sectored colonies (Figure 1). In this case, we posit that the weak staining and phase variation might explain the absence of detection of CPS from broth cultures (Table 1).

In contrast, colonies from the other two CPS producing strains *M. fermentans* PG18 and *M. moatsii* NCTC10158 were not recognized by any of the antibodies used, suggesting that their capsule is composed of other polysaccharides.

**FIGURE 2:**
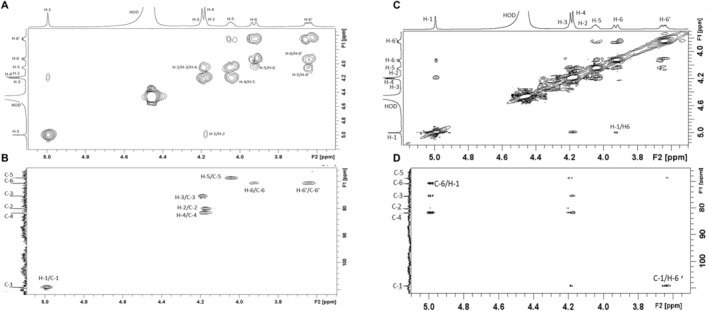
2D 1H/1H COSY NMR spectrum (A), 2D 1H/13C HSQC NMR spectrum (B), 2D 1H/1H NOESY NMR spectrum (C), and 2D 1H/13C HMBC NMR spectrum (D) of CPS purified from *M. fermentans* strain PG18.

Enough CPSs (> 1 mg) from these two strains were successfully purified to proceed to structure determination. The chemical composition and structure of the CPS in *M. fermentans* PG18 were elucidated by high‐performance anion exchange chromatography (HPAEC) analysis (Figure S2) and nuclear magnetic resonance (NMR) spectra (Figure 2). Both analyses revealed that the CPS was composed of glucose. The correlations in the two‐dimensional (2D) NMR ^1^H/^1^H correlation spectroscopy (COSY) (Figure 2A) and ^1^H/^1^H total correlation spectroscopy (TOCSY) spectra were used to attribute various chemical shifts to protons of the glucosyl residues (Table 2). These results were further confirmed by the 2D NMR ^1^H/^13^C heteronuclear single quantum coherence (HSQC) spectrum (Figure 2B), in which the observed connectivities between H‐1 and C‐1 (109.01 ppm), H‐2 and C‐2 (79.99 ppm), H‐3 and C‐3 (75.31 ppm), H‐4 and C‐4 (81.50 ppm), H‐5 and C‐5 (68.56 ppm), and H‐6 or H‐6′ and C‐6 (70.55 ppm) were characteristic of a glucose polymer (Table 2). The H‐1 signal at 4.991 ppm (^1,2^J < 1 Hz), C‐1 at 109.01 ppm, and C‐4 at 81.50 ppm are typical of D‐glucose residues with a β‐furanoside configuration (Alexandersson and Nestor 2022). The H‐6 (3.922 ppm) and C‐6 (70.55 ppm) chemical shifts indicate the presence of a linkage on C‐6 in glucose residues (Gaurivaud et al. 2016). This linkage was confirmed by the presence of connectivities between the H‐1 of glucose (4.991 ppm) and the signal at 3.922 ppm, which corresponded to the H‐6 of glucose in the ^1^H/^1^H nuclear Overhauser effect spectroscopy (NOESY) spectrum (Figure 2C). Similar connectivities were also observed in the ^1^H/^13^C heteronuclear multiple bond correlation (HMBC) spectrum (H‐1/C‐6; 4.991/70.55 ppm) (Figure 2D). These results unambiguously demonstrate that the CPS in *M. fermentans* PG18 is a β‐(1 → 6)‐glucofuranose polymer, which was hereafter named glucofuranan in reference to the β‐galactoglucofurannan from the medicinal macrofungus *Sanghuangporus vaninii* (Cheng et al. 2022).

**TABLE 2:** ^13^C and ^1^H chemical shifts (in ppm) of → 6)‐b‐Glcf‐(1 → CPS in D_2_O at 50°C.

Atoms	1	2	3	4	5	6	6′
^13^C	109.1	79.99	75.31	81.50	68.56	70.55	70.55
^1^H	4.991	4.188	4.201	4.195	4.039	3.922	3.638

In contrast, NMR signals from *M. moatsii* NCTC10158 CPS were not interpretable which could be explained by complex glycoconjuguate molecules such as glycoproteins and/or glycolipids in our extract. Analysis of the purified CPS by SDS PAGE and silver staining evidenced two polypeptides (Figure S3A), suggesting an imperfect purification of the CPS fraction either due to an incomplete proteinase K digestion or a failure to eliminate all residual proteins by trichloracetic acid precipitation. A crude whole protein extract from *M. moatsii* washed cells was analyzed by SDS PAGE and stained by PAS. No high molecular weight smear characteristic of polysaccharides was evidenced but two bands, one of ~80 kDa and another of ~10 kDa, were marked that could be glycoproteins and glycolipids, respectively (Figure S3B). These two structures could be responsible for the nonspecific CPS and EPS signals detected in broth culture. Detailed in silico screening for glycosyltransferases in *M. moatsii* NCTC10158 genome revealed a total of three synthases together with eight other glycosyltransferases belonging to the GT2 or GT4 family (Figure S3C). This exceptional repertoire of GTs contrasts with the situation in other species—for instance, *M. agalactiae* 14628 or *M. fermentans* PG18 have only one synthase and two other GTs as predicted in CAZy database—and might explain the difficulties we encountered in purifying and identifying the CPS produced by *M. moatsii* NCTC10158.

### 2.2 In Silico Prediction of Glucan, Galactan, and Glucofuranan Biosynthesis Pathway in *M. iowae* 695, *M. bovigenitalium* 51080, *M. bovis* F11436, and *M. fermentans* PG18

**FIGURE 3:**
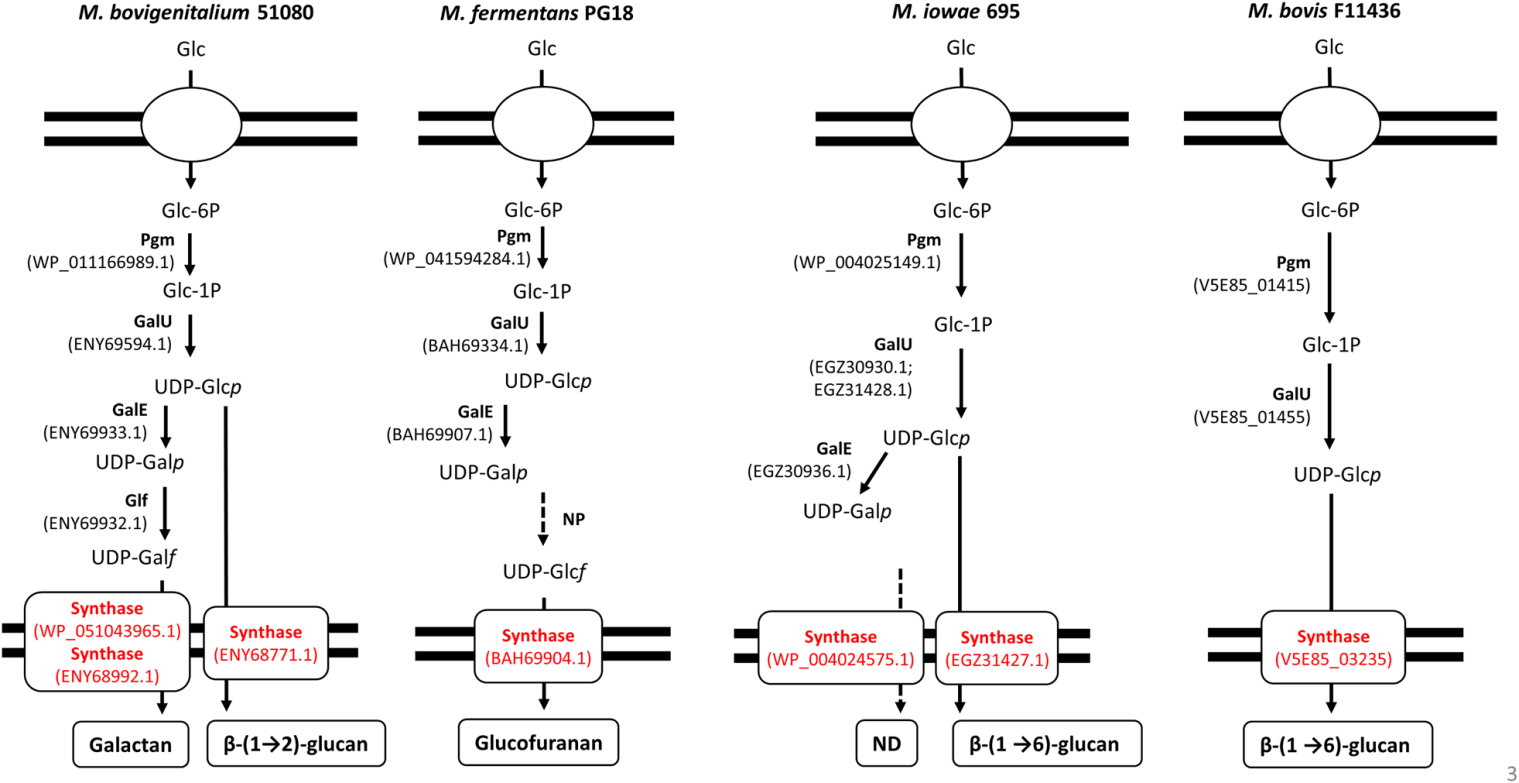
Predicted biosynthesis pathways of glucan, galactan, and glucofuranan secreted by *M. bovigenitalium* 51080, *M. fermentans* PG18, *M. iowae* 695, and *M. bovis* F11436. Predicted synthases are shown in red. ND, not detected; NP, not predicted; Glc, glucose; Galp, galactapyranose; Galf, galactofuranose; Glcf, glucofuranose; GalU, UTP glucose‐1P uridylyltransferase; GalE, UDP‐glucose 4 epimerase; Glf, UDP‐galactopyranose mutase; pgm, phosphoglucomutase. GenBank accession number of enzymes are indicated into brackets. Dotted arrows indicate uncharacterized enzyme or product.

Anabolic pathway leading to glucan and galactan biosynthesis in *Mycoplasma* has been already described for *M. agalactiae* and *M. mycoides*, respectively (Bertin et al. 2013; Gaurivaud et al. 2016). Enzyme sequences involved in *M. agalactiae* glucan and *M. mycoides* galactan synthesis were used to identify their homolog in *M. iowae*, *M. fermentans*, *M. bovis*, and *M. bovigenitalium* by BLASTp. Phosphoglucomutase (pgm) and UTP glucose‐1P uridylyltransferase GalU involved in the synthesis of UDP‐glucose, the monomer unit used for glucan synthesis, were identified in each species. Similarly, GalE, the UDP‐glucose 4 epimerase, able to convert UDP‐glucose into UDP‐galactose was present in all species except *M. bovis* F11436 and the UDP‐galactopyranose mutase Glf, catalyzing the conversion of UDP‐galactopyranose into UDP‐galactofuranose, the building block of galactan, was retrieved only for *M. bovigenitalium* 51080 (Figure 3).

Homologs of synthases were searched using one‐to‐one alignments with the two model synthases in mycoplasmas, that is, *M. mycoides* galactan synthase (CAE76760.1) and *M. agalactiae* glucan synthase (EIN15433.1) (Table 3). The synthase (V5E85_03235) predicted for *M. bovis* strain F11436 (genome accession number JBANDN000000000) showed 79% AA identity (coverage 98%) with the glucan synthase EIN15433 of *M. agalactiae* strain 14628. Interestingly, the genetic environment of both synthases is very similar (Figure S4A) except for a 14 nucleotides insertion in the intergenic region upstream of *M. bovis* synthase gene (Figure S4B).

**TABLE 3:** Amino acid identity between synthases predicted for *M. iowae* 695, *M. bovigenitalium* 51080, *M. bovis* F11436, and *M. fermentans* PG18 with the two model galactan and glucan synthases from *M. mycoides* subsp. *mycoides* PG1 (CAE76760.1) and *M. agalactiae* 14628 (EIN15433.1), respectively.

Species	Strain	Synthases	Amino acid identities % (coverage %) with	
			Galactan synthase CAE76760.1^a^	Glucan synthase EIN15433.1^a^
*M. iowae*	695	WP_004024575.1	No significant similarity	25 (38)
		EGZ31427.1	23 (36)	**36 (91)**
*M. bovigenitalium*	51080	ENY68771.1	No significant similarity	**42 (83)**
		WP_051043965.1	**34 (69)**	23 (52)
		ENY68992.1	**34 (87)**	24 (27)
*M. bovis*	F11436	V5E85_03235	22 (62)	**79 (98)**
*M. fermentans*	PG18	BAH69904.1	28 (93)	19 (27)

^a^ GenBank accession number.

Amino acid identities > 30% (arbitrary threshold) are shown in bold.

Of two synthases predicted for *M. iowae* 695 (Table 3), the highest amino acid identity and coverage with the *M. agalatiae* 14628 glucan synthase EIN15433 was obtained for EGZ31427.1 (36% AA identity, coverage 91%) and we proposed that it could be responsible for producing the glucan CPS detected in vitro for *M. iowae* 695. Moreover, the corresponding gene harbors a poly (TA) stretch at its 5′ extremity (Figure S5), with a variable number of TA dinucleotides in the two versions of the genome available in public databases (GCA_000227355.2; GCA_009883755.2) (Ghanem et al. 2023; Wei et al. 2012) that could be at the origin of the β‐(1 → 6)‐glucan antigenic variation observed by colony blotting (Figure 1). The second predicted synthase (WP_004024575.1) did not return significant AA identity with any of the two model synthases (Table 3).

Of the three predicted synthases of *M. bovigenitalium* 51080, only ENY68771.1 was homolog to the *M. agalactiae* glucan synthase EIN15433 (42% AA identity, coverage 83%) and could be associated to the β‐(1 → 2)‐glucan production observed in vitro, while the two other synthases (WP_051043965.1 and ENY68992.1) showed only a weak AA identity (34% with a coverage of 69% and 87%, respectively) with the galactan synthase of *M. mycoides* CAE76760.1 but could nonetheless be responsible for the galactan production observed in vitro (Figure 1). The potential role of the WP_051043965.1 synthase in the production of galactan is supported by the presence of a sequence of 10 guanine nucleotides at the beginning of the corresponding gene (Figure S6) that could be responsible for the antigenic variation observed by colony blotting (Figure 1).

A complete pathway leading to glucofuranan biosynthesis has not been described so far in bacteria (Alexandersson and Nestor 2022). UDP‐ or NDP‐ (nucleoside diphosphate) glucofuranose could be used as a glucofuranose donor for glucofuranan polymerization by the synthase of *M. fermentans*. Strain PG18 is able to produce UDP‐glucose and UDP‐galactopyranose, which could be involved in UDP‐glucofuranose synthesis (Figure 3). The synthase from *M. fermentans* PG18 showed no significant homology with the galactan or glucan model synthases (Table 3) but with a synthase from *Clostridia* (Figure S7). The corresponding gene is located in a genomic region potentially originating from *Clostridia* and flanked by transposase genes (Figure S7). Of the three other genes belonging to this region two are pseudogenes coding for the amino‐terminal and carboxy‐terminal parts of a protein showing homology with 2‐phospho‐L‐lactate transferase (CofD family). The last gene in this region showed 52% amino acid identity (coverage 99%) with an NDP nucleotidyl transferase (Figure S7) and thus could also be a candidate for UDP‐glucofuranose synthesis. Further experimental proof will be required to decipher the biosynthesis pathway as well as the presence of a similar transposon in other *M. fermentans* strains.

### 2.3 Diversity of Polysaccharide Synthases in Mycoplasmas

In an effort to expand our inventory, all the genomes of the *Mycoplasma* genus (170 species, including alternative genus name *Mycoplasmoides*, *Malacoplasma*, *Mycoplasmopsis*, *Mycoplasma*, and *Metamycoplasma*) available in public databases (NCBI and CAZy, accessed on February 29, 2024) were screened for synthases and GalU, GalE, as well as Glf enzymes (Table S2). Thirty‐seven putative synthases from 28 *Mycoplasma* species spread within various hosts (humans, ruminants, poultry, reptiles, horses, monkeys, dogs) were predicted using both the CAZy database and deepTMHMM for prediction of GT2 with transmembrane domains. Remarkably, no synthase was predicted in species invading swine, such as *M. hyopneumoniae*, *M. flocculare*, and *M. hyorhinis* (in agreement with our in vitro observations, Table 1).

Homologs of motifs known to be of importance in the synthesis of the homopolysaccharides as described in the GT2 family β glucan synthases (Oehme et al. 2019) were searched by multiple sequence alignment for all the 37 synthases with the cellulose synthase subunit A of *Cereibacter sphaeroides* (Figure S8). Those were motifs (i) interacting with divalent cations acting as cofactors (DxD) or UDP‐sugar (DD, KAG, and FxVTxK motifs), (ii) involved in catalysis (ED motif), or in holding the growing glycan chain in the active site (QxxRW) and in di‐c‐GMP (Bis‐(3′‐5′)‐cyclic dimeric guanosine monophosphate) binding PilZ domain (RxxxR and DxSxxG). First of all, synthases from *Mycoplasma* lacked the two motifs of the PilZ domain (Figure S8). The glucan synthases previously described in *M. agalactiae*, *M. mycoides* subsp. *capri*, *M. leachii*, and *M. capricolum*, as well as those described here in *M. iowae*, *M. bovis*, and *M. bovigenitalium* shared several motifs (DD, DxD, KAG, ED, and FxVTxK) (Figure S8). Regarding the galactan and glucofuranan synthases, the KAG motif was not found, the DxD and QxxRW motifs were conserved compared to those of the cellulose synthase but other GT motifs showed different amino acid sequence context (Figure S8).

All synthases had three to six TMH as predicted by DeepTMHMM or four to seven by TMHMM2.0 (Table S2, Figure S8). However, the homology modeling program SWISS‐MODEL (Waterhouse et al. 2018) predicted for galactan, glucans, and glucofuranan synthases a common three‐dimensional structure, that is, a cytoplasmic domain, three helices that run parallel to the membrane (named interface helices, IFs) and a transmembrane channel made of three to six TMHs (Figure S9). The main difference is that the channel is composed of three TMHs for the galactan and glucofuranan synthases and six TMHs for the glucan synthases (Figure S9). This confirmed our previous suggestion to use the number of TMH to differentiate glucan versus galactan synthases (Gaurivaud et al. 2016) and further extend the model to glucofuranan synthases. Of note, the TMHs predicted by TMHMM2.0 or deepTMHMM correspond to both those involved in the channel structure and IF in the SWISS‐MODEL. DeepTMHMM is more accurate for TMH prediction than TMHMM2.0 (Table S2) and more useful than SWISS‐MODEL as modeling could not be obtain for all synthases.

**FIGURE 4:**
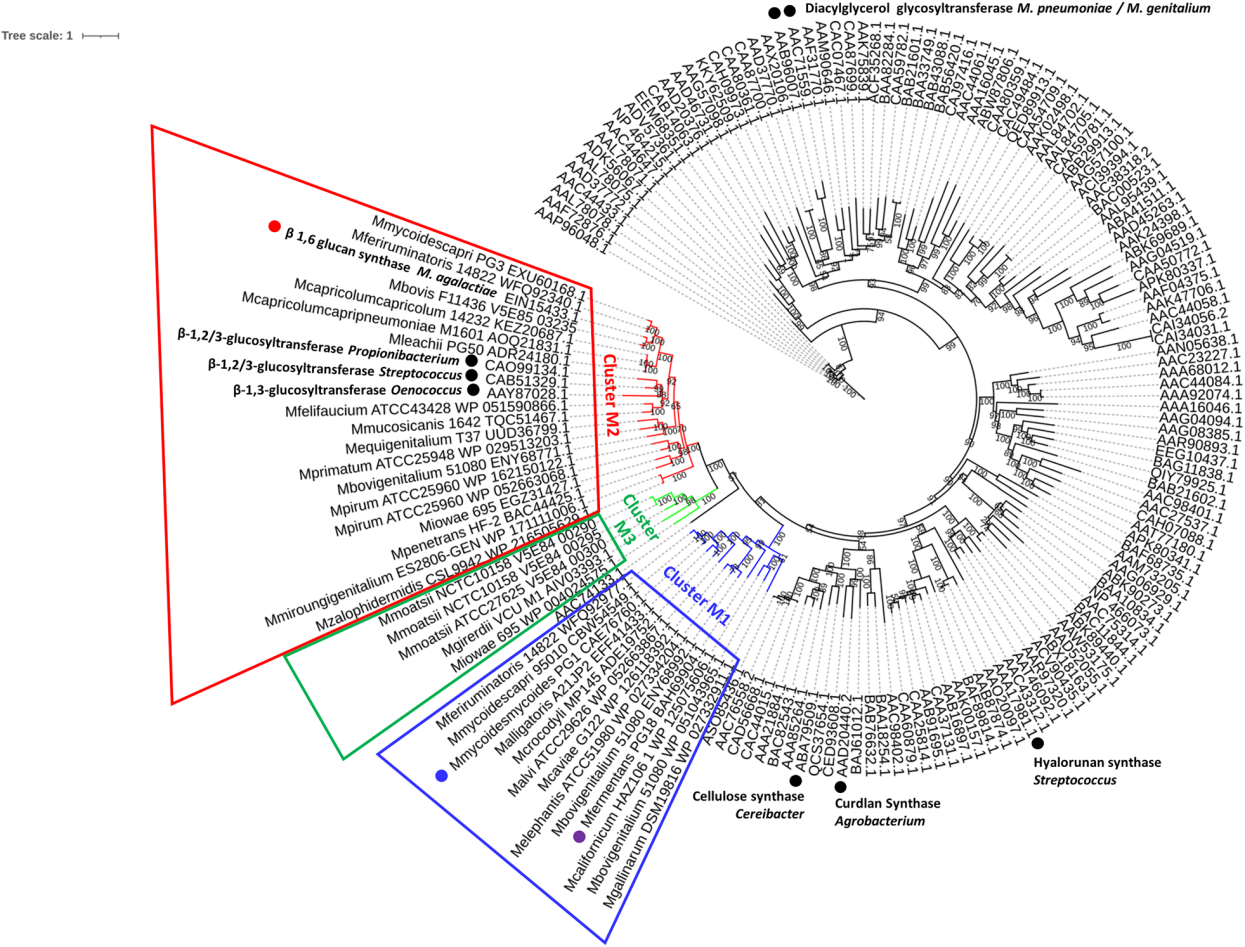
Refined phylogenetic tree of the bacterial GT2 family and mycoplasma synthases. Unrooted 1000‐bootstrap maximum likelihood phylogeny of characterized GT2 family glycosyltransferases extracted from the CAZy database including several synthases Mycoplasma spp. with additional mycoplasma synthases predicted from this work. The two model synthases, *M. mycoides* subsp. *mycoides* β‐(1 → 6)‐galactofuranose (galactan) synthase CAE76760, *M. agalactiae* EIN15433 β‐(1 → 6)‐glucopyranose synthase, and *M. fermentans* β‐(1 → 6)‐glucofuranose (glucofuranan) synthase BAH69904 are indicated by a blue, red, and purple dot, respectively. Some bacterial well‐characterized, representative GT2 enzymes are indicated by black dots (ABA79509.1 cellulose (β‐(1 → 4)‐glucopyranose) from *Cereibacter sphaeroides*; AAD20440.2 curdlan UDP‐Glc: β‐1,3‐glucan synthase (CrdS) from Agrobacterium sp.; AAA17981.1 hyaluronan synthase 1 (HasA) from *Streptococcus pyogenes*; CAO99134.1 (1 → 3, 1 → 2)‐beta‐d‐glucopyranose synthase from *Propionibacterium freudenreichii* subsp. *shermanii*; CAB51329.1 β‐1,2/3‐glucosyltransferase from *Streptococcus pneumoniae*; AAY87028.1 β‐1,3‐glucan synthase from *Oenococcus oeni*). Two GT2 from *Mycoplasma* spp. that are not synthases are also shown with black dots: AAC71559.1 UDP‐Glc: diacylglycerol β‐1,3‐glucosyltransferase from *Mycoplasma genitalium*; AAB96007.1 UDP‐Gal/Glc: β‐Gal‐1,2‐diacylglycerol β‐1,6‐glycosyltransferase from *Mycoplasma pneumoniae*). Enzymes and species corresponding to the accession numbers are listed in Table S3 for bacteria and Table S2 for mycoplasmas.

Phylogenetics has been previously used to predict the enzymatic function of members of the different plant and bacteria GT2 sub‐families (Little et al. 2018; Oehme et al. 2019). Hence, we developed a similar approach and generated a phylogenetic tree using all synthases retrieved from the *Mycoplasma* genus as well as all the GT2 glycosyltransferases with characterized product from bacteria that are listed in the CAZy database (Figure 4, Table S3, http://www.cazy.org/GT2_characterized.html). Mycoplasma synthases clustered into three distinct clusters, very far away from the glycoglycerolipid glycosyltransferase from *M. genitalium* and *M. pneumoniae* that catalyze the addition of one or two glucose or galactose to a glycerolipid and therefore are involved in glycolipids synthesis (Andres, Martinez, and Planas 2011; Klement et al. 2007). Cluster M1 gathers the *M. mycoides* (subsp. *mycoides* and subsp. *capri*) galactan synthases, the glucofuranan synthase from *M. fermentans* as well as several other predicted mycoplasmas synthases (Figure 4). Any mycoplasma species of the M1 cluster provided they also express GalU, GalE, and glf are in theory able to produce a galactan—this could be the case for *M. alligatoris* and *M. crocodyli*, two reptile pathogens or a glucofuranan for species without GalE and glf such as *M. gallinarum* and *M. callifornicum* (Table S1). Cluster M2 includes previously described synthases from *M. agalactiae*, *M. leachii*, *M. capricolum*, and those proposed in the present paper (*M. bovis* V5E85_03235; *M. iowae* EGZ 31427.1 and *M. bovigenitalium* ENY68771.1) but also synthases from a large repertoire of human or animal mycoplasmas (Figure 4, Table S1). The closest non‐mycoplasma hits are three glucan synthases from the Gram‐positive bacteria *Streptococcus*, *Oenococcus*, and *Proponibacterium* (Figure 4). The third cluster, M3, is close to M2 and gathers synthases for which the final product has not been identified yet, from *M. iowae 695*, *M. moatsii* NCTC10158, and Candidatus *M. girerdii* VCU_M1, a human mycoplasma not yet cultivated but considered a strict endosymbiont of *Trachomonas vaginalis* (Bailey et al. 2023).

## 3 Discussion

The purification of polysaccharides secreted by mycoplasmas has been hindered for years by the complexity of growth media (Bertin et al. 2013), poor survival in synthetic medium, and potential phenotypic variation in the expression of the biosynthetic pathway resulting in either many contaminants or small amounts of polysaccharides (Gaurivaud and Tardy 2022). The method previously developed for *M. agalactiae* (Gaurivaud et al. 2016) was successful here for purifying and characterizing a new capsular polysaccharide produced by *M. fermentans* PG18. This CPS was further identified as a glucofuranan (β‐(1 → 6)‐glucofuranose) homopolymer unbranched and decorated with no other chemical substituents as for other mycoplasmal homopolysaccharides. To our knowledge, this is the first glucofuranose homopolymer described in bacteria. Lipopolysaccharides from the plant pathogen *Erwinia amylovora* do contain glucofuranose units but those are not homopolymers and are certainly short chains (Ray et al. 1987). In Eukaryotes, glucofuranose polymers have previously been reported in the plant *Ophiopogon japonicas* (Wang et al. 2012) and in the fungus *Sanghuangporus vaninii* (Cheng et al. 2022). Polysaccharide secretion by *M. fermentans* PG18 had previously been suspected because of the ability of the strain to form a biofilm (Awadh et al. 2021). The role of this particular polysaccharide in biofilm formation and in host interaction has yet to be deciphered, in comparison with the galactan (β‐(1 → 6)‐galactofuranose), with known immunomodulatory activities (Totte et al. 2015). Antibodies against glucofuranan will be helpful in detecting this polymer in other strains or mycoplasma species.

For *M. moatsii* NCTC10158 using the same purification technique, we demonstrated the biosynthesis of a complex polymer containing polysaccharides among other things. The presence of glycoconjuguates with membrane‐anchoring lipids or peptides, in association with the presence of 11 predicted GTs, prevented us from deciphering the chemical structure of the polysaccharide moiety by NMR. Similar glycoconjugate structures, that is, a glycoprotein with a high molecular weight, susceptible to proteinase treatment, and a glycolipid with a low molecular weight, had been observed in the human pathogen *M. penetrans* GTU‐54‐6A1 (Neyrolles et al. 1998).

By using a battery of available specific antibodies in colony blotting assays, we were further able to enlarge the number of strains and species able to produce galactan, β‐(1 → 2)‐glucan or β‐(1 → 6)‐glucan. Phase variation resulting in sectored colonies using immunoblotting assays were evidenced for *M. iowae* I695 β‐(1 → 6)‐glucan synthesis but also for *M. bovigenitalium* 51080 β‐(1 → 2)‐glucan and galactan synthesis. As suggested previously, phase variation might allow to adapt to diverse environments (Gaurivaud et al. 2016). Overall our results point toward a rather “universal” production of CPS in *Mycoplasma* species colonizing varied animals such as ruminants, primates, and poultry, and expressing a synthase. Nonetheless, for each species here only one strain was screened and an important intraspecies variation in CPS expression has already been demonstrated for *M. agalactiae*, *M. capricolum*, and *M. mycoides* (Bertin et al. 2015; Gaurivaud et al. 2016). In conclusion, the role of CPS production or phase variation at the species level might be approached with caution.

In previous work, we evidenced structural differences among synthases from seven *Mycoplasma* species/subspecies and for which polysaccharides have been identified, as the number of TMH was useful to orient toward a glucan or galactan synthesis (Ambroset et al. 2017; Baby et al. 2023; Gaurivaud et al. 2016). The relation between the number of TMHs and glucan or galactan secretion was confirmed here for *M. bovis* F11436, *M. bovigenitalium* 51080, and *M. iowae* 695. However, glucofuranan and galactan synthases could not be differentiated using the number of TMHs and synthases with six TMHs were observed for the β‐(1 → 2)‐glucan and β‐(1 → 6)‐glucan synthases as well as other synthases with unknown activities (*M. moatsii* V5E84_00300 and *M. iowae* WP_004024575.1). We further proposed to compare synthases as well as an extended set of glycosyltransferases by focusing not only on some structural features but on their whole amino acid sequence using phylogenetics on the basis of what was classically proposed for predicting function of diverse glycosyltransferases (Chang et al. 2023; Oehme et al. 2019; Spiers et al. 2023). The generated phylogenetic tree clearly separated galactan/glucofuranan synthases (cluster M1) from glucan synthases (cluster M2) and the five synthases with unknown activity (cluster M3). However within each cluster, glycosyltransferase structural motifs were highly diverse. This prevents a straight forward prediction of the synthases specificity only from in silico data and highlights the necessity to conduct functional studies on the model of what we did for *M. agalactiae* (Gaurivaud et al. 2016).

Synthases were predicted from the genome of numerous species (Table S2) with the exception of the two swine species *M. hyopneumoniae* and *M. hyorhinis* (CAZy database), for which in our hands no CPSs/EPSs were detected in vitro. This contrasts with the fact that polysaccharides containing biofilms had been evidenced in the swine pathogen *M. hyopneumoniae* (Tassew et al. 2017). However, biofilm production was shown to vary greatly between different *M. hyopneumoniae* isolates (Wu et al. 2022). The absence of EPS/CPS detection in our experimental conditions might therefore be due to the screened isolate, to culture conditions (liquid culture versus biofilm formation set‐up) or to the synthesis of glycoconjugates not detectable by our methods. For instance, the use of specific antibodies to detect polysaccharides was clearly limiting as only a few antibodies are currently available. Staining with lectins could be an alternative for detecting polysaccharides for which antibodies are not available (Daubenspeck et al. 2009). A sucrase‐dependent pathway (Schmid 2018) could be hypothesized for these two swine species, because glycosyl hydrolases GH13, similar to those involved in cyanobacterial starch biosynthesis (Suzuki et al. 2015), were predicted in the CAZy database (*M. hyopneumoniae* strain NCTC10127, VEU65556.1; *M. hyorhinis* strain NCTC10121, VEU57715.1). Synthases were also not predicted for *M. pulmonis* and *M. genitalium*, suggesting atypical polysaccharide production by these two pathogens as synthases and other classical bacterial pathway such as the canonical ABC transporter‐ or Wzx/Wzy‐dependent pathway (Schmid 2018) were not retrieved in silico in mycoplasmas (Rendueles et al. 2018). Overall, our results showed an unexpected diversity of polysaccharides and homopolysaccharide synthases in *Mycoplama* and suggested other, yet undeciphered, pathways for polysaccharide synthesis and release in these minimal bacteria. Furthermore, the presence in the genomes of many other GT2—up to eight here for *M. moatsii*—suggests that oligosaccharidic decoration of lipid and peptide moieties might also be of importance for mycoplasmas.

## 4 Experimental Procedures

### 4.1 *Mycoplasma* Strains and Culture

*Mycoplasma* strains were kept in a collection at ANSES. Ruminants and human mycoplasma isolates were cultivated in PPLO (PleuroPneumonia‐Like Organism) medium (Indicia technologies) at 37°C under 5% CO_2_. Avian mycoplasma isolates were propagated in FM4 medium (Freundt 1983) at 37°C without CO_2_ and porcine isolates in Friis medium at 37°C (Friis 1975). The concentration of viable bacteria was determined by (i) colony counting (CFU/mL) on agar plates seeded using serial dilutions in a liquid medium and (ii) estimating color‐changing unit (CCU)/mL as described previously (Rodwell and Whitcomb 1983).

### 4.2 Detection of CPSs and EPSs From In Vitro Culture

Stationary‐phase liquid cultures of mycoplasmas were numerated (T0, Table 1) and centrifuged for 20 min at 12,000× *g* 18°C, and the pellets were washed with phosphate buffer saline (PBS) and resuspended and incubated in CMRL medium (Life Technologies) for 72 h at 37°C. CFUs were determined after incubation (T72). Cultures were centrifuged at 14,000× *g* for 1 h at 4°C. Cell‐free polysaccharides were extracted from the cell‐free supernatant and CPSs from the cell pellet as described previously (Bertin et al. 2013; Gaurivaud et al. 2016). Briefly, cells were resuspended with PBS buffer and incubated for 4 h at 37°C with proteinase K 100 μg/mL. The cell suspension was further incubated overnight at 37°C with 100 μg/mL proteinase K, 100 U/mL Dnase, 500 μg/mL RNase, and 0.5% (w/v) SDS. After incubation, residual proteins were precipitated with 10% (w/v) fresh trichloracetic acid for 1 h at 4°C then centrifuged for 1 h at 14,000× *g*, 4°C. Polysaccharides in the supernatant were precipitated with 10 volumes of cold acetone and incubated at −20°C for 1 week. After 1 h of centrifugation at 14,000× *g*, 4°C, acetone was removed and the pellet was air‐dried and resuspended in Milli‐Q (mQ) water. The concentration of polysaccharides was determined by the phenol‐sulfuric method (Dubois et al. 1951) against a glucose standard curve. The limit of quantification of the phenol/sulfuric assay in our experimental conditions had previously been estimated at 0.8 μg of glucose/mL (Gaurivaud et al. 2016).

### 4.3 Colony Blotting

Monoclonal antibodies against β‐(1 → 2)‐glucopyranose and against galactan (β‐(1 → 6)‐galactofuranose) were kindly provided by Dr. Lucia Manso‐Silvan (CIRAD) (Bertin et al. 2015), and rabbit polyclonal antibodies anti β‐(1 → 6)‐glucopyranose (Gaurivaud et al. 2016) were used to detect the corresponding polysaccharides by colony blotting as described previously (Bertin et al. 2015, 2013; Gaurivaud et al. 2016). Briefly, colonies on PPLO agar plates were transferred onto nitrocellulose membrane and nonspecific binding on the membrane was blocked by incubation during 30 min at room temperature with 5% horse serum in PBS. Membrane were incubated with antibodies in 5% horse serum PBS 2 h at room temperature and washed 3 times with PBS 0.05% tween 20 and with PBS. Peroxidase‐conjugated antibodies were incubated 2 h at room temperature, and the membranes were washed as above. The presence of antibodies was revealed with 4‐chloro‐1‐naphtol giving a blue/purple staining. Ponceau red was used to stain colonies or part of colonies not recognized by antibodies. Ponceau red counterstaining was omitted when (purple) staining with antibodies was weak. Specificity of each antibody batch was checked in our experimental conditions by testing isolates known to produce galactan (*M. mycoides* subsp. *capri* strain 95010), β‐(1 → 6)‐glucopyranose (*M. agalactiae* 14628), or β‐(1 → 2)‐glucopyranose (*M. capricolum* subsp. *capricolum* F10190) (Figure S1).

### 4.4 SDS‐PAGE Characterization of Glycoconjugates

CPS extracts were separated on SDS PAGE (Bio‐Rad, AnykD miniprotean gel, SDS tris glycine buffer). After electrophoresis, polypeptides were detected by coomassie blue or silver staining (Sigma Proteosilver kit) and glycoconjugates were revealed using the periodic acid‐Schiff method (GlycoPRO kit from Sigma). Crude extracts from *M. moatsii* NCTC10158 were prepared from PPLO late exponential medium culture; then, cells were pelleted, washed three times with PBS buffer by centrifugation at 12,000× *g* for 30 min at 4°C, and suspended in PBS buffer. Protein concentration was measured using the Pierce BCA protein assay kit (Thermofisher scientific).

### 4.5 Composition and Structure of CPSs

CPSs were extracted directly from PBS‐washed mycoplasmas cultivated in PPLO medium as previously described (Gaurivaud et al. 2016). Air‐died polysaccharides were resuspended and dialyzed against regularly renewed sterile ultrapure water for 48 h using 3.5‐kDa‐cutoff dialysis tubing (Spectrum Laboratories).

The monosaccharide components were determined after hydrolysis of CPSs (1 mg) with 4 M CF_3_COOH (100°C for 4 h). Aliquots of the extract were analyzed by high‐performance anion exchange chromatography (HPAEC) equipped with a pulsed amperometric detector (Dionex ICS 3000 system) and four 50‐mm Propac PA1 pre‐columns (Dionex) followed by a CarboPak PA 1 column at 30°C. A multi‐step gradient elution was performed as follows: 0–25 min, 90% H_2_O and 10% NaOH 160 mM; 25–34 min, 100% NaOH 200 mM; 35–50 min 90% H_2_O and 10% NaOH 160 mM at a flow rate of 1 mL/min. Peak analysis was performed using Chromeleon software, version 7.0.

For nuclear magnetic resonance (NMR) spectroscopy, CPSs were exchanged twice with 99.9% D_2_O (Euriso‐top), dried under a vacuum then dissolved in 99.96% D_2_O (3 mg/0.5 mL). ^1^H NMR spectra were recorded at 50°C on a Bruker Avance 500 spectrometer equipped with a BBI probe (5‐mm sample diameter) and Topspin 1.3 software. ^1^H NMR spectra were accumulated using a 30° pulse angle, a recycle time of 1 s, and an acquisition time of 2 s for a spectral width of 3000 Hz for 32‐K data points using an experimental sequence provided by Bruker. ^13^C NMR experiments were conducted using the same spectrometer operating at 125.48 MHz with 2 s as the relaxation delay. The two‐dimensional (2D) ^1^H/^1^H correlation spectroscopy (COSY), ^1^H/^1^H total correlation spectroscopy (TOCSY) with mixing time of 40 ms to 120 ms, ^1^H/^1^H nuclear Overhauser effect spectroscopy (NOESY) with mixing time of 300 ms, ^1^H/^13^C heteronuclear single quantum coherence (HSQC), and ^1^H/^13^C heteronuclear multiple bond correlation (HMBC) with long‐range delay of 120 ms spectra were acquired with standard pulse sequences provided by Bruker. Chemical shifts were expressed in parts per million (ppm) relative to 3‐trimethylsilylpropionate‐*d*_*4*_ (TSP‐*d*_*4*_).

### 4.6 Genome Sequencing, Assembly, and Annotation of *M. moatsii* NCTC10158 and *M. bovis* F11436

We sequenced the *M. moatsii* NCTC10158 genome as the first version published in the NCBI has been removed because of contaminations. Genomic DNA was extracted from two 72 h cultures of 2 mL each, using an Epicentre kit by Lucigen. The concentration of the gDNA (6 μg at 30–500 ng/μL for Oxford Nanopore Technology (ONT) DNA sequencing and 100 ng at 5 ng/μL for Illumina sequencing) and quality (OD260/280 ≥ 1.8 and OD260/230 ≥ 2) were checked using a Nanodrop spectrophotometer (Thermo Fisher) and a Qubit fluorimeter (DNAds BR kit, Thermo Fisher), respectively. Both Illumina (paired‐150 end libraries, NovaSeq sequencers) and ONT (MinION technology) sequencing technologies were used to sequence genome isolates; this sequencing was outsourced (PGTB, INRAE, Bordeaux; iGenSeq, ICM). Raw data quality control (FASTQ files for both short and long reads), hybrid assembly genome, and annotation were performed according to previously published protocols and parameters (Ambroset et al. 2022). Briefly, the raw data were trimmed for quality (cutoff set at Q25 for Illumina reads and Q16 for ONT reads) and length (up to 50 pb for Illumina reads and 10,000 pb for ONT reads). Unicycler 0.4.8 (Wick et al. 2017) was used for hybrid assembly and annotated by PGAP in the NCBI website. Assembly statistics and genome completeness were computed using QUAST 5.0.2 (Gurevich et al. 2013) and Busco score with Busco 4.1.4 (Seppey, Manni, and Zdobnov 2019). All these bioinformatics tools and pipelines were run using the IFB Core cluster resource (https://www.france‐bioinformatique.fr/cluster‐ifb‐core).

### 4.7 Prediction and Analysis of Enzymes Involved in Polysaccharide Synthesis From Genomic Data

Enzymes involved in UDP‐sugar synthesis (GalU, GalE, and Glf) were identified using BLASTp with default parameters and proteins already identified from *M. mycoides* subsp. *mycoides* strain PG1 (GalU CAE76762.1, *Gal*E CAE77587.1, *Glf* CAE77593.1 / CAE77586.1) or *M. agalactiae* strain 14628 (GalU EIN15406.1) as a query (Ambroset et al. 2017; Bertin et al. 2015, 2013; Gaurivaud et al. 2016). Putative synthases were retrieved from the CAZy database (Drula et al. 2022); proteins annotated as GTs in the CAZy database for the *Mycoplasma* genus (Table S1) were analyzed by TMHMM2.0 (Krogh et al. 2001) and DeepTMHMM (Hallgren et al. 2022) in order to identify GTs with several TMH, indicating a GT‐membrane anchoring with a transmembrane domain as described for synthases (Low and Howell 2018; Whitney and Howell 2013). The selected proteins were then used as a query to identify other putative synthases from mycoplasma genomes using the BLASTp program with default parameters (Altschul et al. 1997) against the NCBI's reference proteins database. Proteins showing homology with the amino‐terminal or carboxy‐terminal part of putative synthases were also selected, and the presence of a homopolynucleotide inside the corresponding genes was assessed in order to detect a potential reversible frameshift. The impact on the potential frameshift of adding or removing repeated nucleotides was analyzed with OrfFinder (Sayers et al. 2011). The structure of predicted synthases was finally analyzed with deepTMHMM (https://dtu.biolib.com/DeepTMHMM, Hallgren et al. 2022) to predict the number of TMHs; only proteins with TMHs in the amino‐terminal and carboxy‐terminal regions were selected for further analyses.

Multiple sequences were aligned using Clustal Omega (https://www.ebi.ac.uk/Tools/msa/clustalo/) (Sievers and Higgins 2018) then visualized with Mview (Madeira et al. 2024). They were used to identify GT active sites defined for the *Cereibacter sphaeroides* (former *Rhodobacter sphaeroides*) cellulose synthase subunit A (BcsA, WP_011338158.1).

Predicted secondary and tertiary structures were produced using SWISS‐MODEL with default options (Bienert et al. 2017; Waterhouse et al. 2018). Only predicted models for the full sequence of the query proteins were selected; the parameters of the structure predictions are shown in Figure S9. All modeling was performed by the end of July 2024. Models were visualized and compared using the SWISS‐MODEL structure assessment interface (www.https://swissmodel.expasy.org/).

### 4.8 Phylogenetics

Phylogenetic analysis were performed as described by Oehme et al. (Oehme et al. 2019). Briefly, full sequence of mycoplasmas synthases and the characterized bacterial GT2 glycosyltransferases retrieved from the CAZy database (http://www.cazy.org/GT2_characterized.html, 142 sequences from bacteria, accession on June 2024) were aligned using Clustal omega (https://www.ebi.ac.uk/jdispatcher/msa/clustalo). The optimal substitution model (VT + F + G4) and tree construction was done by Iqtree (IQ‐TREE 1.6.12 built August 15, 2019). The resulting tree was edited using iTOL online server (Letunic and Bork 2024).

## Author Contributions

**Manon Vastel:** investigation, validation. **Corinne Pau‐Roblot:** investigation, validation, writing – original draft, writing – review and editing. **Séverine Ferré:** investigation. **Véronique Tocqueville:** investigation. **Chloé Ambroset:** investigation. **Corinne Marois‐Créhan:** conceptualization, writing – review and editing. **Anne V. Gautier‐Bouchardon:** conceptualization, funding acquisition, writing – review and editing, writing – original draft. **Florence Tardy:** conceptualization, funding acquisition, writing – original draft, writing – review and editing, supervision, visualization. **Patrice Gaurivaud:** conceptualization, writing – original draft, funding acquisition, writing – review and editing, supervision, visualization.

## Supporting information


Figures S1–S9.

Figure S8.

Table S1.

Table S2.

Table S3.

## Data Availability

The bioproject associated to this study is PRJNA1075496. Nucleotide sequence assemblies were submitted to GenBank and are available under the accession numbers JBANDN000000000 (*M. bovis* F11436) and CP146987 (*M. moatsii* NCTC10158).
