# Supplementary material for: Capsular Polysaccharide Production in Bacteria of the Mycoplasma Genus: A Huge Diversity of Pathways and Synthases for So‐Called Minimal Bacteria
Source: Mol Microbiol. 2024 Oct 30;122(6):866–78. doi: 10.1111/mmi.15325 (PMC11658790; doi:10.1111/mmi.15325)
Supplement: Supplementary file 1 — Figures S1–S9. [file MMI-122-866-s003.pdf]

Capsular polysaccharide production in bacteria of the *Mycoplasma* genus: a huge diversity of pathways and synthases for so-called minimal bacteria

Supplementary information:

Vastel_Supplementary_Figures_REV: supplmentary Figure S1, S2, S3, S4, S5, S6, S7 & S9

Vastel_Supplementary_Figure_S8

Vastel_Supplementay_Table_S1

Vastel_Supplementay_Table_S2

Vastel_Supplementay_Table_S2

FIGURE S1 Colony immunostaining with anti galactan, anti β-(1→6)-glucan and anti β-(1→2)-glucan specific antibodies against *M. mycoides* subsp. *capri* 95010*, M. capricolum* subsp. *capricolum* F10190 and *M. agalactiae* 14628 cultivated on PPLO agar plates and used as control for Figure1. Colonies marked by antibodies are dark purple/grey (positive, P). Colonies not recognized by antibodies are pink due to counterstaining with red Ponceau (negative, N). "S" indicates sectored colonies. Scale bar = 1 mm.

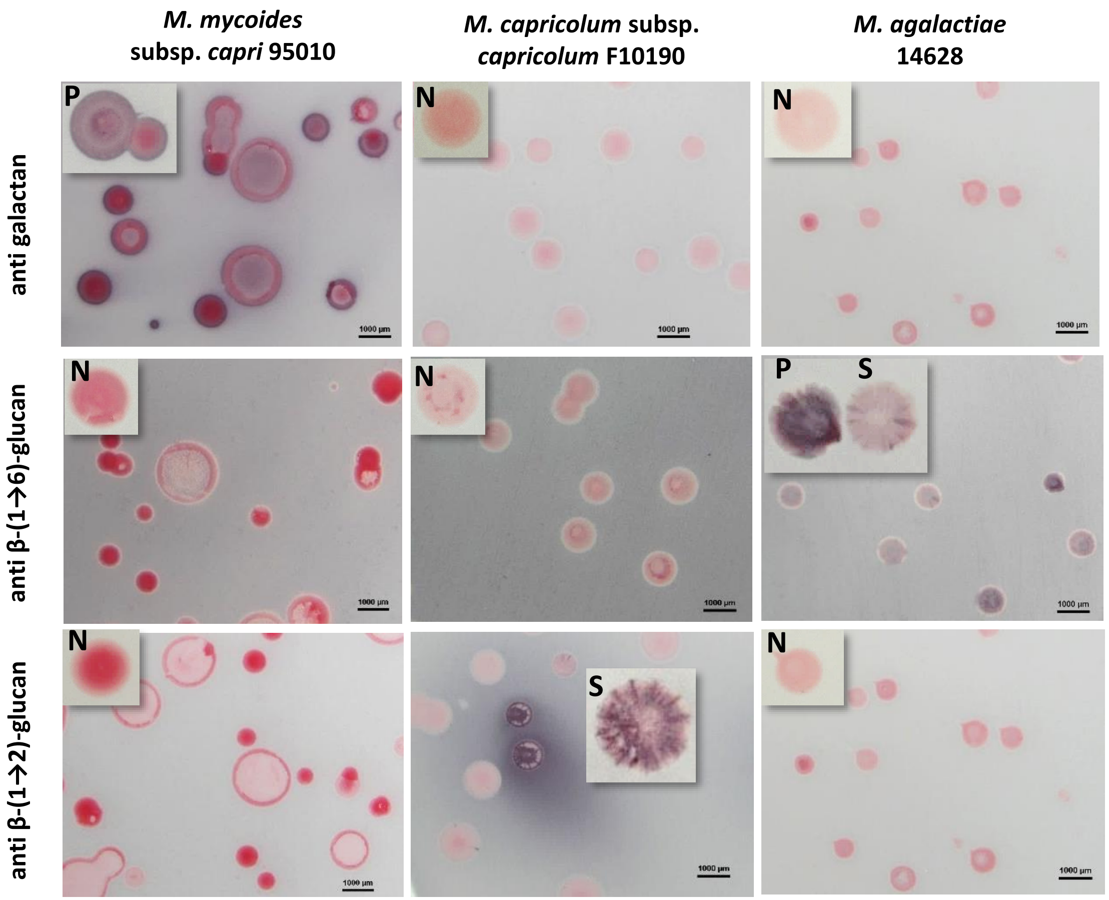

Figure S2 (a): HPAEC-PAD profiling of CPS after TFA hydrolysis; (b): HPAEC-PAD profiling of standard neutrals sugars

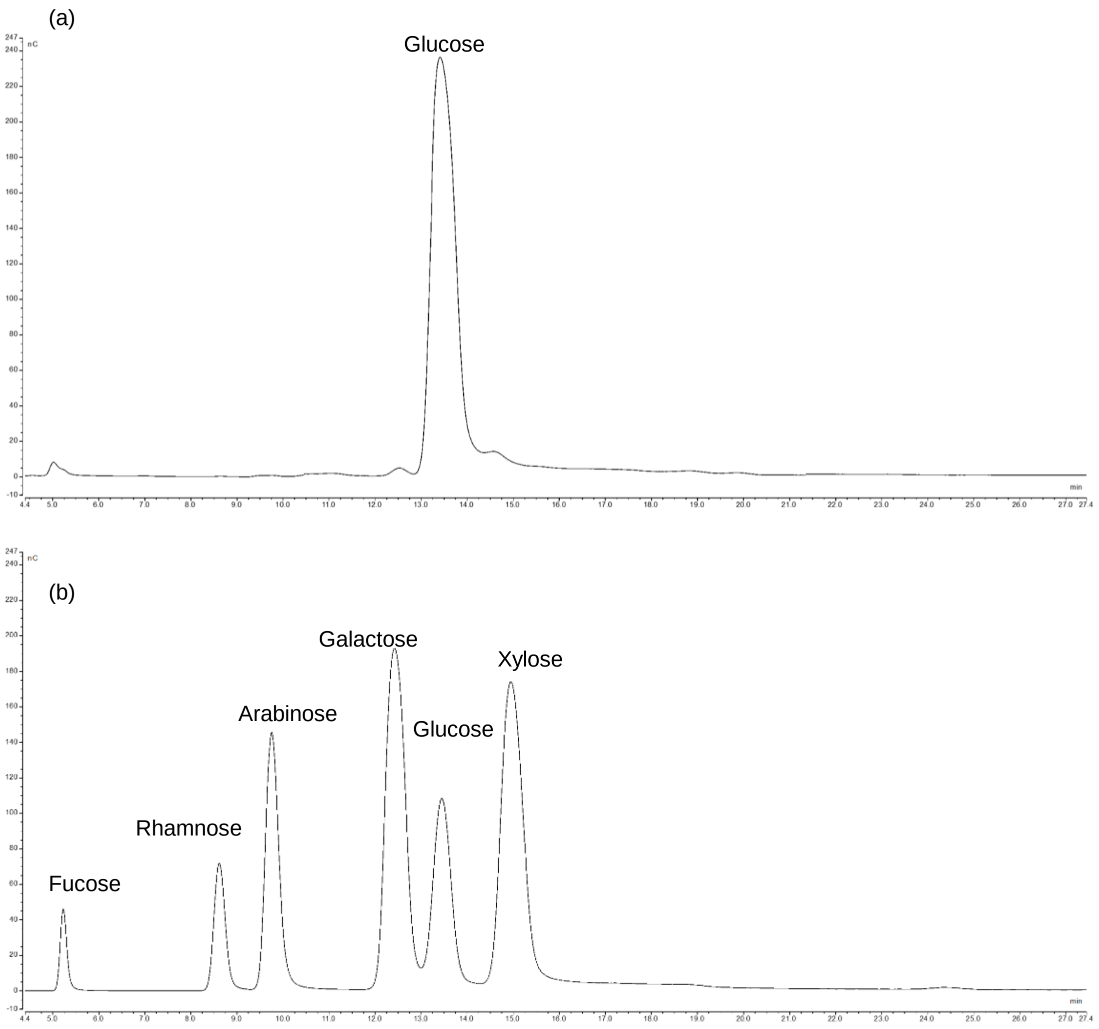

FIGURE S3 A, SDS PAGE of CPS extract (2 µg) from *M. moatsii* NCTC10158 stained by silver staining (lane 2, arrows showed polypeptides revealed by silver staining in the CPS extract). B, whole protein extract (20 µg) of *M. moatsii* NCTC10158 after SDS PAGE, Schiff staining (lane 3, arrows showed glycoproteins or glycolipids revealed by PAS staining) and coomassie blue staining (lane 4). Lane 1, protein ladder in kDa. C, list of GT predicted from the genome of *M. moatsii* strain NCTC10158.

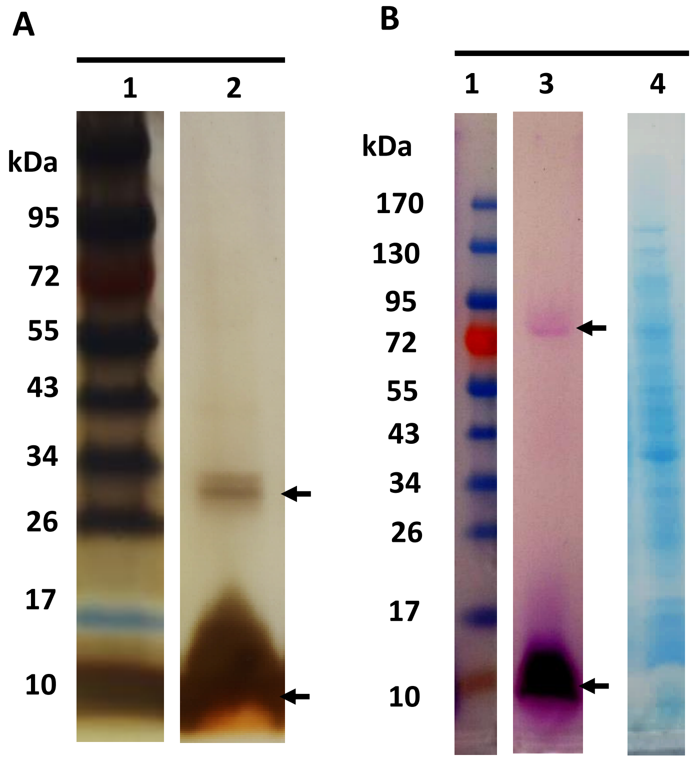

C

M. moatsii NCTC10158 accession number	GT family	Number of transmembrane helices (TMH) predicted by DeepTMHMM
V5E84_00265	GT2	0
V5E84_00290	GT2	6
V5E84_00295	GT2	6
V5E84_00300	GT2	6
V5E84_00475	GT2	0
V5E84_01270	GT2	0
V5E84_02035	GT2	2 at the C-terminus extremity*
V5E84_02695	GT2	0
V5E84_02900	GT2	0
V5E84_02905	GT4	0
V5E84_02910	GT4	0

*This structure differs from the synthase structure for which the GT domain is surrounded by TMH.

FIGURE S4 A, Schematic representation of the locus containing the synthase gene in *M. agalactiae* strain 14628 and *M. bovis* F11436. **B,** Alignment of the synthase gene intergenic region (in black) from *M. bovis* strain F11436 and *M. agalactiae* strain 14628. The start codon of the synthase gene is underlined and the beginning of the synthase gene is in green. The end of the upstream gene is in purple (the stop codon is underlined). The nucleotides stretch inserted in the intergenic region of strain F11436 is framed.

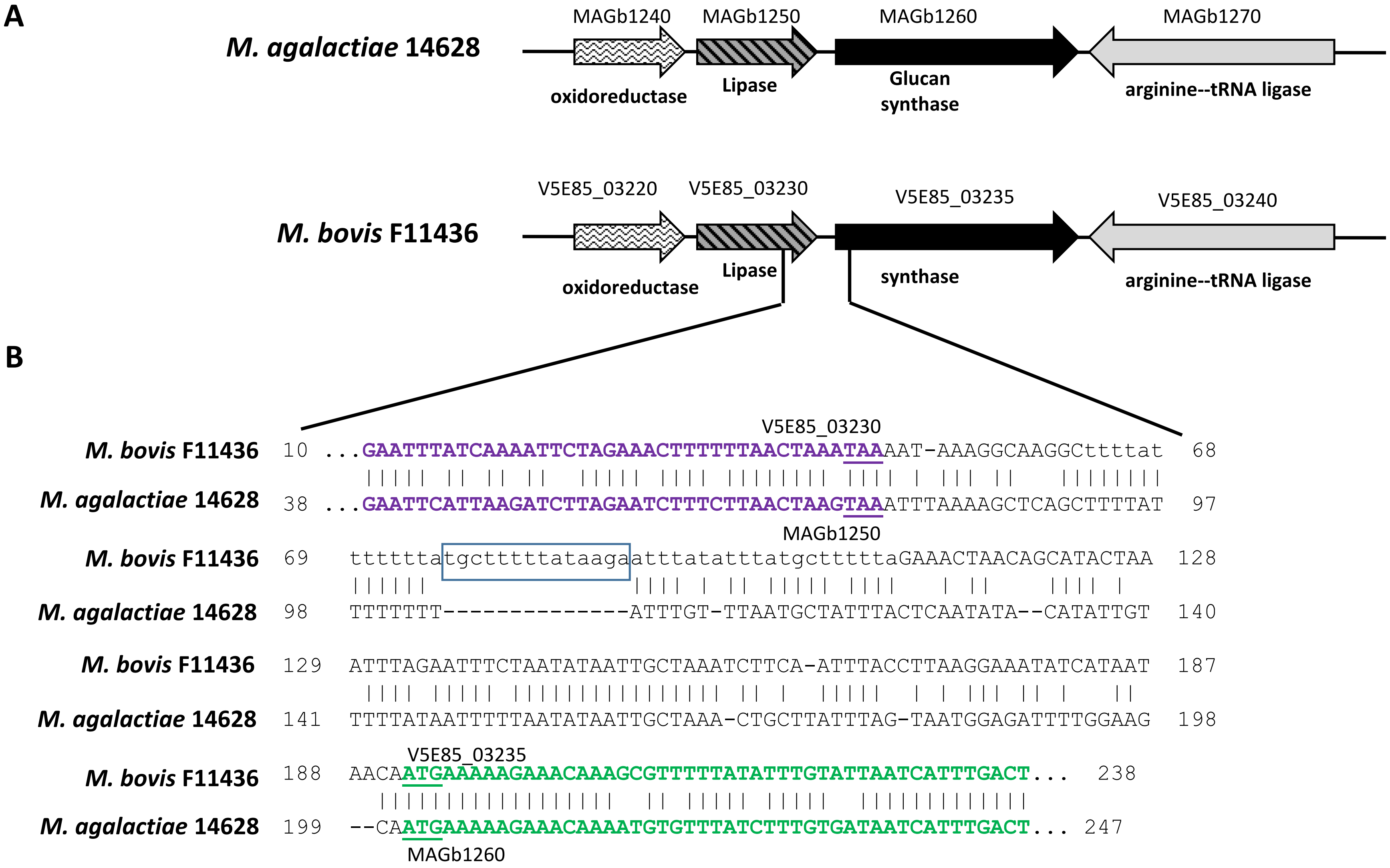

FIGURE S5 TA-repeats (bold) in *M. iowae* 695 synthase EGZ31427.1 gene as published by Wei et al. in 2012. The truncated protein missing the 116 first amino acids WP_229502539 as published by Ghanem et al. in 2023 is underlined.

EGZ31427.1

1 ATGGCTAAAAAACAAAAAATGTATTTTTCTAATAACAA**TATATAT**
M A K K Q K M Y F S N N N I Y

46 **ATATATATATATATATATA**GTTGAATAATAATTTTATTACTTTGA
I Y I Y I Y S W I I I L L L W

91 GTCGGACTAATAATTTTTTCATATTGAATTAACATTTATAGTCAA
V G L I I F S Y W I N I Y S Q

136 TGAAAATTTACAACCAATACATATTCTCCAATAGTTTGTAAAATT
W K F T T N T Y S P I V C K I

181 CTATTAGTTTTTAACTTTATATTTGTTAGTTCATTATACTGTTCG
L L V F N F I F V S S L Y C S

226 TCAATAAAAAATTTTATTTTCACTCTTGCATATTTATTTTTTTAT
S I K N F I F T L A Y L F F Y

271 AAAAGGAAAAATTCTAGATATGTATATACTGTAATAGAAAATAAA
K R K N S R Y V Y T V I E N K

316 TCTTTAGATATCATTCTCAAAAAAAATCCAATGGTCTTTGTTTTA
S L D I I L K K N P M V F V L

361 TACACAACATGTAATGACTTTGATAGCATTGCACTTAAAAAATCT
Y T T C N D F D S I A L K K S

406 TTATATCAAACATATCAAAATTGTAAATTTTTTATATTAGATGAT
L Y Q T Y Q N C K F F I L D D

451 TCTAATGATAGTAAATACAAGGATGAAATTGATAAATTTGCTTAT
S N D S K Y K D E I D K F A Y

496 GAATATAATATTGAAGTTATAAGAAGAAAAAATAGAAAAGGTTTT
E Y N I E V I R R K N R K G F

.
.
.

1486 TCTTTTATATATATTCCAAATATTAACTTTTTCACATATGTTTGA
S F I Y I P N I N F F T Y V W

1531 GTAATTATACTAATAATACCTATGTTTTTAACGGTTCCTTTAACC
V I I L I I P M F L T V P L T

1576 CTAATGTCTAATATTAAACTAAAAAATAAAAATCATGAAATGGAG
L M S N I K L K N K N H E M E

1621 AATAAATATGTCATCTAA
N K Y V I *

WP_229502539

1 ATGGTCTTTGTTTTATACACAACATGTAATGACTTTGATAGCATT
M V F V L Y T T C N D F D S I

46 GCACTTAAAAAATCTTTATATCAAACATATCAAAATTGTAAATTT
A L K K S L Y Q T Y Q N C K F

91 TTTATATTAGATGATTCTAATGATAGTAAATACAAGGATGAAATT
F I L D D S N D S K Y K D E I

136 GATAAATTTGCTTATGAATATAATATTGAAGTTATAAGAAGAAAA
D K F A Y E Y N I E V I R R K

181 AATAGAAAAGGTTTTAAAGCAGGTAATATAAATAATTTTCTAATG
N R K G F K A G N I N N F L M

226 AATAGAAATGATTATGATTATTTTGTTTTACTAGATTCAGATGAA
N R N D Y D Y F V L L D S D E

271 ATAATTCCAAAAGATTATATTTTAAAAGTTCTACCATATTTTTCC
I I P K D Y I L K V L P Y F S

316 AATAAAAAAGTTGGTATTGTTCAATGTAATAACAAGTGCAATAGG
N K K V G I V Q C N N K C N R

361 CAAAATAATTATTTTGATTATTTAGGATCATATGTACATAATACA
Q N N Y F D Y L G S Y V H N T

406 CAATGAAATACTGAATATGTAGTCAGACATTCACTTGGTGTTGTT
Q W N T E Y V V R H S L G V V

451 AATTGTTGTGGTCACGGAGCAACCATAAGTAAAAAAATGTATTTA
N C C G H G A T I S K K M Y L

496 GATGTTGGTGGTTTCCCAGAATTAATATTAGAAGATTGAGCCTTA
D V G G F P E L I L E D W A L

541 ACTCTTAATGGATTAAAGTTTGGTTATGAAACTGTGTATGCACCT
T L N G L K F G Y E T V Y A P

586 GAAATTGTATGTTGAGAAGAATTCCCAACAAATTATCTGGCATTT
E I V C W E E F P T N Y L A F

631 AAAAAAAGAAATTATAGATGAACACAAGGAGGTGTTCAATGTTTT
K K R N Y R W T Q G G V Q C F

676 TCAAAAATAGGATGAAAATTACTTTTTTTCAATTCTTGTCCTTTT
S K I G W K L L F F N S C P F

721 TTTAGAAGACTTGATTTATTATTAACGCAAACAAATTATTTTATA
F R R L D L L L T Q T N Y F I

766 AGTATAGGATCATTAATAATAGTTTTAAGTACACTATCAATTCTT
S I G S L I I V L S T L S I L

811 GCACCAATGGGTTTTAGATTTAGATATGAAAATTGATATGTAGCT
A P M G F R F R Y E N W Y V A

856 GTTACTTTATTTTTCGCAATAATACCATTATTGAATTGCTTTATA
V T L F F A I I P L L N C F I

901 TATTATTTAGGAAAAGTAAATTTTTTTAAACTAATTGTCATTCTA
Y Y L G K V N F F K L I V I L

946 CTATTTTTTTATATATTATATGCATCTTTGTTTGTATCAGTAATA
L F F Y I L Y A S L F V S V I

991 ATTTCAATATTTGAAGCACTAATAGGAAAAAAGTTAAATTTTGTA
I S I F E A L I G K K L N F V

1036 GTAACACCAAAAAAAGAAACTAAAAGAATATCTTTTATCGACGCC
V T P K K E T K R I S F I D A *

FIGURE S6 PolyG stretch (bold, underlined) in *M. bovigenitalium* 51080 synthase WP-051043965.1 gene resulting in a STOP codon and hence in pseudogeneisation. Adding one G (+1G) to the polyG stretch would restore a complete synthase gene.

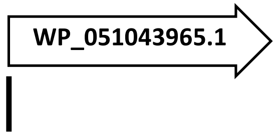


```
  1 ATGCAAATATTTTTAATGTGGTTCAATATATCGATGTTGATATTT
     M  Q  I  F  L  M  W  F  N  I  S  M  L  I  F

 46 TCCCTTTTATTCCTATTTTTAAATATCTTGTATTTTGTTGTTGGA
     S  L  L  F  L  F  L  N  I  L  Y  F  V  V  G

 91 TTCATATTGAGGATATTTAAAAATAAAAAGAATAGTTTAATGTGT
     F  I  L  R  I  F  K  N  K  K  N  S  L  M  C

136 ACTATGCACAAATTTGCGATTTTAATCCCTGCTCGAAACGAAAGT
     T  M  H  K  F  A  I  L  I  P  A  R  N  E  S

181 AGTGTGATTGCGAATTTAATTAAGTCACTTAAATGTCAAAATTAT
     S  V  I  A  N  L  I  K  S  L  K  C  Q  N  Y

226 CCATCTGATTTATTTGATATTTATGTAATTGCAGACAATTGCACT
     P  S  D  L  F  D  I  Y  V  I  A  D  N  C  T

271 GATAATACAAAAGAAATTGCTGACCAACAGGGTGCAATTGTTATT
     D  N  T  K  E  I  A  D  Q  Q  G  A  I  V  I

316 GAAAGAAATGATGCTCAAAACATCGGCAAAGGCTATACTTTGGAT
     E  R  N  D  A  Q  N  I  G  K  G  Y  T  L  D

361 TATGCAATAAAGCATATTAACCAATTAAGACGGGGGGGGGGCAGC
     Y  A  I  K  H  I  N  Q  L  R  R  G  G  G  S

406 TATCTAATTCATTAG
     Y  L  I  H  *
```

10G

+ 1G

```
   1 ATGCAAATATTTTTAATGTGGTTCAATATATCGATGTTGATATTT
      M  Q  I  F  L  M  W  F  N  I  S  M  L  I  F

  46 TCCCTTTTATTCCTATTTTTAAATATCTTGTATTTTGTTGTTGGA
      S  L  L  F  L  F  L  N  I  L  Y  F  V  V  G

  91 TTCATATTGAGGATATTTAAAAATAAAAAGAATAGTTTAATGTGT
      F  I  L  R  I  F  K  N  K  K  N  S  L  M  C

 136 ACTATGCACAAATTTGCGATTTTAATCCCTGCTCGAAACGAAAGT
      T  M  H  K  F  A  I  L  I  P  A  R  N  E  S

 181 AGTGTGATTGCGAATTTAATTAAGTCACTTAAATGTCAAAATTAT
      S  V  I  A  N  L  I  K  S  L  K  C  Q  N  Y

 226 CCATCTGATTTATTTGATATTTATGTAATTGCAGACAATTGCACT
      P  S  D  L  F  D  I  Y  V  I  A  D  N  C  T

 271 GATAATACAAAAGAAATTGCTGACCAACAGGGTGCAATTGTTATT
      D  N  T  K  E  I  A  D  Q  Q  G  A  I  V  I

 316 GAAAGAAATGATGCTCAAAACATCGGCAAAGGCTATACTTTGGAT
      E  R  N  D  A  Q  N  I  G  K  G  Y  T  L  D

 361 TATGCAATAAAGCATATTAACCAATTAAGACGGGGGGGGGGGCAG
      Y  A  I  K  H  I  N  Q  L  R  R  G  G  G  Q

 406 CTATCTAATTCATTAGAATATGATGCTTTTATTGTGTTTGATGCT
      L  S  N  S  L  E  Y  D  A  F  I  V  F  D  A

 451 GACAATGTGGTTGATAAAGATTTTTTACACTACATCAATATTGAG
      D  N  V  V  D  K  D  F  L  H  Y  I  N  I  E

 496 TATTCAAAAGGATACAAAATAATTACATCATACAGAATGCCTAAA
      Y  S  K  G  Y  K  I  I  T  S  Y  R  M  P  K

 541 AATTACAATTCATCGTGGGTTGCAGCAATCTCTGGATTAATGTAC
      N  Y  N  S  S  W  V  A  A  I  S  G  L  M  Y

 586 ATTCGTGAATCAGTATTTGTTAATAGACCAAAAAAAGCTCTTAAT
      I  R  E  S  V  F  V  N  R  P  K  K  A  L  N

 631 ATAAGTTGTTCGATATCTGGCACAGGATTTTTAGTTGACTCAAAT
      I  S  C  S  I  S  G  T  G  F  L  V  D  S  N

 676 ATTTTAAAAGATGGTTGAAAATACCATTTACTAACTGAGGATATT
      I  L  K  D  G  W  K  Y  H  L  L  T  E  D  I

 721 CAACTATCATCAGATTTAGTTTCAAAGGGTCAAAAAATAGGTTAT
      Q  L  S  S  D  L  V  S  K  G  Q  K  I  G  Y

 766 GCTAAAGATGCTATAACATATGATGAGCAGCCGACAGATTTAAAA
      A  K  D  A  I  T  Y  D  E  Q  P  T  D  L  K

 811 ACATTTTTTAGACAACGAACTCGCTGAGCTAAAGGTTTTCTACAA
      T  F  F  R  Q  R  T  R  W  A  K  G  F  L  Q

 856 GTTTTTAGAAAATATTCTGGAAAACTATTTTTAAAATCATTCACA
      V  F  R  K  Y  S  G  K  L  F  L  K  S  F  T

 901 AGTATATCTGCATTTGATATATTTTCATTCATTTTCCCAACTAGT
      S  I  S  A  F  D  I  F  S  F  I  F  P  T  S

 946 TTAATTTTTATTCTTTCATTTTTGGTTAATTTGTCATTTGCGATT
      L  I  F  I  L  S  F  L  V  N  L  S  F  A  I

 991 TTTGGATATTCAACTTCTAACATTGCTCTAGGAAATTATGCAATT
      F  G  Y  S  T  S  N  I  A  L  G  N  Y  A  I

1036 ATTAATTTAACTATCGGAATTAGTTTAACACTTTTAACTCCTTTT
      I  N  L  T  I  G  I  S  L  T  L  L  T  P  F

1081 GTTTTTGGATTAATAACAACTTTAACCGAGTACAAAAGAATCAAA
      V  F  G  L  I  T  T  L  T  E  Y  K  R  I  K

1126 GCTTCTTTTATAAAAAAGATTTGATACTCAATTCTGTTTCCGATT
      A  S  F  I  K  K  I  W  Y  S  I  L  F  P  I

1171 GCATTTTTCATTTACTTAGCAGCTTTTTTAACTGCGCTATTAAAG
      A  F  F  I  Y  L  A  A  F  L  T  A  L  L  K

1216 AAAAAAGTAGTTTGGAAACCTATTAAACATTCAGACACAAAAGAT
      K  K  V  V  W  K  P  I  K  H  S  D  T  K  D

1261 ATAGTTCAAATGTAG
      I  V  Q  M  *
```

11G

FIGURE S7: Schematic representation of the genomic region carrying the glucofuranan synthase gene (Mbio-0639) in *M. fermentans* PG18 strain. Blastp results are shown under the genetic organization. ID, protein identity percentage; coverage, cov; MBIO-0XXX, locus tag for *M. fermentans* PG18; Nt, NH_2 terminal part; Ct, COOH terminal part; NTP, nucleoside triphosphate.

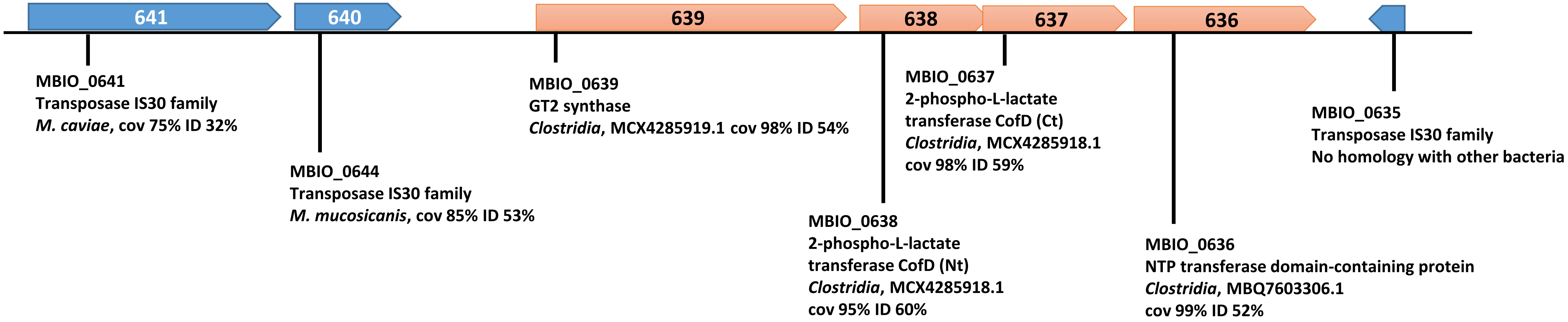

FIGURE S9 Swiss-model prediction of the 3D-structures of mycoplasmal synthases (side view). Transmembrane helices involved in the channel are showed in olive, and those acting as interface helices in blue. The glycosyltransferase domains are in grey, the membrane surface in hatched grey.

M. mycoides **subsp.** ***mycoides strain*** **PG1 galactan synthase CAE76760.1**

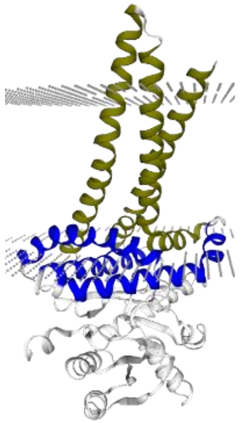

M. fermentans strain **PG18 glucofuranan synthase BAH69904.1**

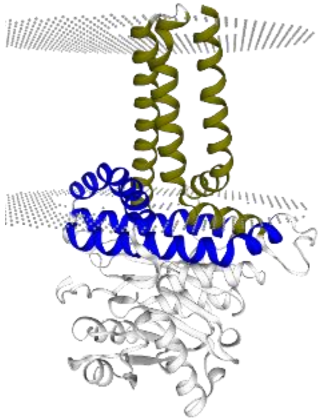

M. bovigenitalium strain **51080 galactan synthase ENY68992.1**

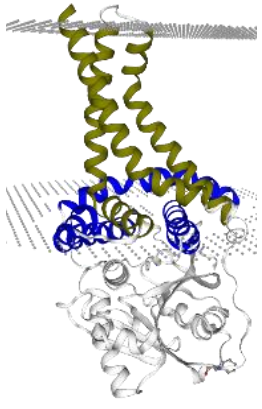

M. agalactiae **strain 14628 β-(1→6)-glucan synthase EIN15433.1**

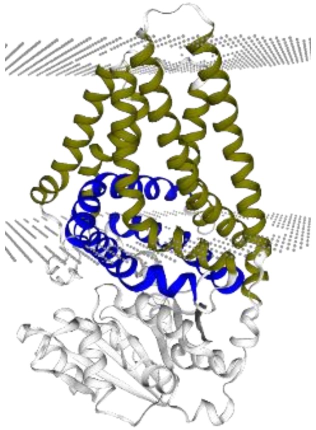

M. iowae **strain 695 synthase EGZ31427.1**

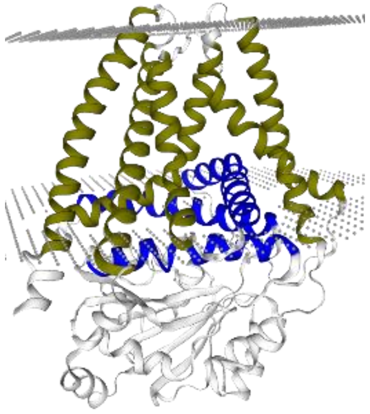

**Query**				**Swiss model**			
**Species**	**Strains**	**CPS**	**Synthase accession number**	**GMQE (1)**	**Model**	**Sequence identity**	**Coverage**
M. mycoides subsp. mycoides	PG1	β 1,6 galactofuranose	CAE76760.1	0.90	AlphaFold DB model of A0A7U0XN17 (gene: unknown, organism: unknown)	94.43%	100%
M. capricolum subsp. capricolum	14232	β 1,2 glucopyranose	KEZ20687.1	0.91	AlphaFold DB model of A0A0C3A2I3_MYCCA (gene: A0A0C3A2I3_MYCCA, organism: Mycoplasma capricolum subsp capricolum)	99.43%	100%
M. agalactiae	14628	β 1,6 glucopyranose	EIN15433.1	0.89	AlphaFold DB model of I5D6X4_MYCAA (gene: I5D6X4_MYCAA, organism: Mycoplasmopsis agalactiae 14628)	100%	100%
M. fermentans	PG18	β 1,6 glucofuranose	BAH69904.1	0.92	AlphaFold DB model of A0A449AMC2 (gene: unknown, organism: unknown)	100%	100%
M. iowae	695	β 1,6 glucopyranose	WP_004024575.1	0.70	AlphaFold DB model of A0A6P1LDR6_MALIO (gene: A0A6P1LDR6_MALIO, organism: Malacoplasma iowae 695)	100%	100%
M. iowae	695	undertermined	EGZ31427.1	0.83	AlphaFold DB model of A0A6P1LBF6_MALIO (gene: A0A6P1LBF6_MALIO, organism: Malacoplasma iowae 695)	98.89%	100%
M. bovigenitalium	51080	β 1,6 galactopyranose	ENY68992.1	0.93	AlphaFold DB model of A0A1L7MZB3_9MOLU (gene: A0A1L7MZB3_9MOLU, organism: Mycoplasmopsis bovigenitalium)	97.24%	100%
M. bovigenitalium	51080	β 1,2 glucopyranose	ENY68771.1	0.84	AlphaFold DB model of N9VBJ4_9MOLU (gene: N9VBJ4_9MOLU, organism: Mycoplasmopsis bovigenitalium 51080)	100%	100%
M. bovis	F11436	β 1,6 glucofuranose	V5E85_03235	0.90	AlphaFold DB model of A0A4U0WA74 GT2 Mycoplasma bovis 8790	93.28%	100%

(1) Global model quality estimate

M. bovis **strain F11436 β-(1→6)-glucan synthase V5E85_03235**

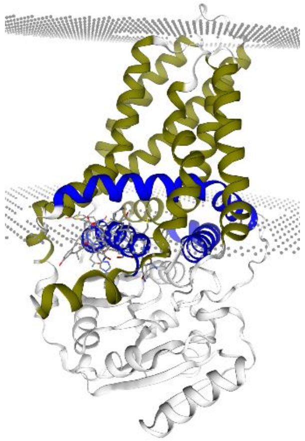

M. bovigenitalium strain **51080 β-(1→2)-glucan synthase ENY68771.1**

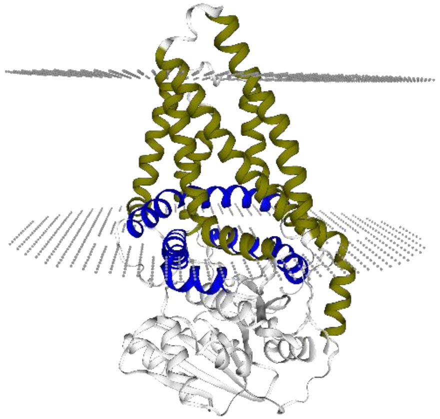

NOTE: modelling of the *M. bovigenitalium* synthase WP_051043965.1 (424 AA) was not obtained for the full length of the protein (coverage 66% (147-424), GMQE 0.62).
