# Supplementary material for: Capsular Polysaccharide Production in Bacteria of the Mycoplasma Genus: A Huge Diversity of Pathways and Synthases for So‐Called Minimal Bacteria
Source: Mol Microbiol. 2024 Oct 30;122(6):866–78. doi: 10.1111/mmi.15325 (PMC11658790; doi:10.1111/mmi.15325)
Supplement: Supplementary file 2 — Figure S8. [file MMI-122-866-s001.pdf]

FIGURE S8 Clustal Omega alignment between the cellulose synthase subunit A (BcsA WP_011338158.1) from *Cereibacter sphaeroides* and synthases from mycoplasmas. Names of the BcsA motifs involved in cation binding (DxD), catalysis (ED), UDP sugar interaction (DD, KAG, FxVTxK), polysaccharide coordination (QxxRW) and PilZ domain (RxxxR, DxSxxG) involved in di-C-GMP binding are indicated above the sequences and identical amino acids are underlined. Sequences are shaded according to the clusters colors in Figure 4 (blue for cluster M1, red for M2 and green for M3) and TMH domains are highlighted in yellow.

```
                                                   1         .         .         .         .         :         .         .         .         .         1         .         . 120
 1 Mfermentans_PG18_BAH69904.1                     ---------------------------------------------------------------------------------------------------------------MVMVYQNVV
 2 Mbovigenitalium_51080_ENY68992.1                --------------------------------------------------------------------------------------------M----------------QQLVLATRIIT
 3 Mcaviae_G122_WP_126118392.1                     --------------------------------------------------------------------------------------------M----------------EKLNLSTVIIG
 4 Melephantis_ATCC51980_WP_027334204.1            -------------------------------------------------------------------------------------------ML----------------ENLILSSNIIN
 5 Malvi_ATCC29626_WP_052663867.1                  ---------------------------------------------------------------------------------------------------------------MTNTINTLF
 6 Mferiruminatoris_14822_WFQ92977.1               -----------------------------------------------------------------------------------------MIIN----------------HQLNTATTVFF
 7 Mmycoidescapri_95010_CBW54549.1                 ------------------------------------------------------------------------------------------MIN----------------QHLNIVTNVFF
 8 Mmycoidescapri_95010_CBW53832.1                 ------------------------------------------------------------------------------------------MLN----------------SQLNTTTTVFF
 9 Mmycoidesmycoides_PG1_CAE76760.1                ------------------------------------------------------------------------------------------MLN----------------SQLNTTTTVFF
10 Malligatoris_A21JP2_EFF41433.1                  -------------------------------------------------------------------------------------------------------------MELKLAFYIFF
11 Mcrocodyli_MP145_ADE19752.1                     -------------------------------------------------------------------------------------------------------------MTLFYITNILF
12 Mgallinarum_DSM19816_WP_027332911.1             ------------------------------------------------------------------------------------------MQW----------------DTSKLSFTFFW
13 Mcalifornicum_HAZ106_1_WP_125075606.1           ------------------------------------------------------------------------------------------------------------------MLNFLW
14 Mbovigenitalium_51080_WP_051043965.1            ------------------------------------------------------------------------------------------------------------------MQIFLM
15 Csphaeroides_BcsA_WP_011338158.1                ----MTVRAKARSP----------LRVVPVLLFLLWVALLVPFGLLAAAPVAPSAQGLIALSAVVLVALLKPFADKM-VPRFLLLSAASMLVMRYWFWRLFETLPPPALDASFLFALLLF
16 Miowae_695_WP_004024575.1                       ---MFNSRARYLNN---------VFNIVTKIFFIFF---SLLLS----------ILFILN-----------AFNVINIIPDYLYI-------DKNNPWTVLL------------------
17 Mgirerdii_VCU_M1_AIV03393.1                     ---MFQIKARDKIN---------KFNFVKKIVN-IF---SLLILSVITT-----SLLFAF-----------L----I---------------TGNK--HLVF------------------
18 Mmoatsii_NCTC10158_V5E84_00300                  ---MFHLKTRDEYR---------KKMYWIRLSFQFF---TLLFFVV-F------ILNTIY-----------VFNLVN---------------ISQSFFKIFL------------------
19 Mmoatsii_NCTC10158_V5E84_00290                  ---MFQLKSRNSYT---------KKVFYVRLCASLF---FVPYFIF-WN-----ILLLIY-----------ISNTNI---------------ISKSFFDTFN------------------
20 Mmoatsii_NCTC10158_V5E84_00295                  ---MFQLKSRNSYT---------KKVFYVRLCASLF---FVPYFIF-WN-----ILLLIY-----------ISNTNI---------------ISKSFFDTFN------------------
21 Mmiroungigenitalium_ES2806-GEN_WP_171111006.1   ----MKLTDENNIKYKSH---ILDRKKYFILSIILRILVFAGSLSLGWL----IVYKLS------------NYNTLI--AD-----------L-PSDWR---LIV---------LFVFVM
22 Mzalophidermidis_CSL9942_WP_216505629.1         ----MKLTDENNIQYKSY---RLNRKKHFILSIILRILVFASSLSLSWL----IVYKLA------------NYNALI--SD-----------L-PSDWR---LIV---------LFSFLM
23 Mequigenitalium_T37_UUD36799.1                  ------MQTKIISKSK----KLTKKPTAYLIAVLIF---LCLSSLYLYF----AINNVI------------DLLWNF--NS-----------YTSN--QKIKTCA---------IVIFIF
24 Mbovigenitalium_51080_ENY68771.1                --------------MQ----NIKKSPFIFIISIIVA---CFLGAIGGYF----VFQ--I------------IYQNVI--NN-----------YGLSKINDVKIIF---------SIIFFC
25 Mprimatum_ATCC25948_WP_029513203.1              --------------MK----KLVKQRKVFFISIVLF---FMISAFLFYL----SSNSII------------TYFKNE--NF-----------FNADTWTIVKQIS---------MTILFV
26 Mfelifaucium_ATCC43428_WP_051590866.1           MSEKTEKTYKTGSLLK-------KNSLFYWVLLIVW---LLLSALFIYI----YITKIN--SF--TFAQKDPFNNVI--KD-----------------TTSRRAA---------AIIFIT
27 Mmucosicanis_1642_TQC51467.1                    ---MFKDKKNPLMILR-------KNSSFYWLMIIIW---SSLIIMLTYF----FVTRISYVSL--DIGKKDPFGNII--TA-----------------TKARVVS---------AKIFLV
28 Mleachii_PG50_ADR24180.1                        --------------MK-------KNSSVYSLIIGFW---TICNCLIIAS----VATSSV-----------MLFIRQS--DL-----------------KLYNKIL---------IMIVLW
29 Mcapricolumcapricolum_14232_KEZ20687.1          --------------MK-------KKSNVYSLIIGFW---TICNCLIIAS----VVTSSV-----------MLFIRQS--DL-----------------KLYNKIL---------IVIILW
30 Mcapricolumcapripneumoniae_M1601_AOQ21831.1     --------------MK-------KKSNVYSLIIGFW---TICNCLIIAS----VLTSSA-----------MLFIRQT--DL-----------------KLYNKIL---------IVIILW
31 Magalactiae_14628_EIN15433.1                    --------------MK-------KKQNVFIFVIIIW---LILCSAIIVP----LILKFI-----------NLFTVPN--NY-----------------SLAIKIV---------IVILLI
32 Mbovis_F11436_V5E85_03235                       --------------MK-------KKQSVFIFVLIIW---LILSSAIIVP----LIFKFI-----------NLFSAEN--TY-----------------SLAVKII---------IVIMLS
33 Mmycoidescapri_PG3_EXU60752.1                   --------------MK-------KHSYLYKWILIIW---FIISLSITIP----LVFKFI-----------HLFNADD--SH-----------------SLTTKII---------IAVLLT
34 Mmycoidescapri_PG3_EXU60168.1                   --------------MK-------KQSLLYKWILVIW---FLVSLAITVP----LVFKLI-----------KLFSVQG--NQ-----------------ALATKIV---------ISILLT
35 Mferiruminatoris_14822_WFQ92340.1               --------------MK-------KQSLLYKWILIIW---FLVSLTITIP----LVFKLI-----------KLFSIQN--GQ-----------------ALATKIV---------ILVLLV
36 Mpirum_ATCC25960_WP_162150122.1                 ------------MQSK-------KANIPYILFFVFW---LIFSGLSFWY----MI----------------VYQWNH--TL-----------------LSVNEVV---------VKIFIV
37 Mpirum_ATCC25960_WP_052663068.1                 --------------MK-------KINAPYILIFVLW---GGFTSLSSWY----LI----------------VFQWEN--TF-----------------FTLQDIV---------VKFFLI
38 Miowae_695_EGZ31427.1                           ------MAKKQKMYFSNNNIYIYIYIYSWIIILLLW---VGLIIFSYWI----NI----------------YSQWKF--TT-----------------NTYSPIV---------CKILLV
39 Mpenetrans_HF-2_BAC44425.1                      ----MKNTKNKFINLKKDKKEKYLYNLIWWITILLW---ILVVILSYYV----NI----------------ILQWEN--TN-----------------FSYSKIA---------TRILIF
```

```
                                                      121         .         .         :         .         .         .         .         2         .        .DD        .         . 240
 1 Mfermentans_PG18_BAH69904.1                             YIVMPIIVAIFL-----IFSFQYWFVLFFGLFAKPVKF-------------KTQT--QKLKYGI---VICARNEEKVIAELVKSIKNSQYDQDKLQTFVIAHNCTDK-----TAEKA---
 2 Mbovigenitalium_51080_ENY68992.1                        YIGIGITTLFTA-----IFFLQI-FYTFFALFIKNKRF-------------NKTN--KFNNHCI---IIPAHNEAHIIGDLIKSLKAMKYPKQYFKVFVVADNCTDN-----TAQVA---
 3 Mcaviae_G122_WP_126118392.1                             YITLGISILFFL-----IFFLQI-AYTFLALFIKNKKF-------------KKTD--VYNNHAI---LIPAHNEGHIIADLVKSLKAMDYPADKFKVFVVADNCTDN-----TADVA---
 4 Melephantis_ATCC51980_WP_027334204.1                    WISLGICILFFL-----IFFLQI-VYTFLPLFIKNKKF-------------EHTN--KYNNHCI---IIPAHNEAHIISDLITSLKSMNYPKDKFKVFVVADNCTDN-----TAEVA---
 5 Malvi_ATCC29626_WP_052663867.1                          IISIVFSLLSFV------FNFFEYFLALIGIFKKRAISL----------RKIDFL--PNKKIAV---LIAARNEENVIKDIVNSLLNQTYNKNFFHIYLVADNCTDN-----TAKIVQKI
 6 Mferiruminatoris_14822_WFQ92977.1                       YLTLVIGIIFML------FLSSDWLFFMF-AYIKNKKKL----------DKYKPK--KNRSFAI---IIPAHNESSVVGKLIDSIKAQKYDG-VIDIYLVADNCNDN-----KKTYNVG-
 7 Mmycoidescapri_95010_CBW54549.1                         YITLVIGIIFML------FLSSDWLFFMF-SYIKNKKKL----------IKYQPK--KNRAFAI---IIPAHNESVVVGKLIDSIKAQKYDG-IIDIYLVADNCTDK-----KKTYNVG-
 8 Mmycoidescapri_95010_CBW53832.1                         YLTLVIGIIFML------LLSSDWLFFMF-AYTKNKKKL----------AKHKPK--KNRSFAI---VIPAHNESMVVGKLIDSIKAQKYDG-VIDIYLVADNCTDN-----RKTYNVG-
 9 Mmycoidesmycoides_PG1_CAE76760.1                        YITLVIGIIFML------LLSSDWLFFMF-AYTKNKKKL----------AKHKPK--KNRSFAI---VIPAHNESMVVGKLIDSIKAQKYDG-IIDIYLVADNCTDN-----RKTYNVG-
10 Malligatoris_A21JP2_EFF41433.1                          WATIGIGLFFFL------LYSLEWKYFF--NSRKKKNIL----------ADYQPK--KLRRFAI---VIPARNESITLKKLIESIQKQNYLG-TIDIYLVADNCTDN-----RQTYNIG-
11 Mcrocodyli_MP145_ADE19752.1                             WVTFIIGIIFFV------IYGLEWIHFF--NSRKNINSL----------NNYTPK--KTRNFAL---IIPARNESVTLSKLINSMQEQKYDG-KIDIYLVADNCTDN-----RMTYNIG-
12 Mgallinarum_DSM19816_WP_027332911.1                     V---FLGITLFLFLIAILFHYFKFQYFFYAFFNRKKAQN----------IFPKAE--KQHYFTY---LIPARDESKVIENLLKSIKNQAYDSNKINVVIGVEDLNDP-----TVKIA---
13 Mcalifornicum_HAZ106_1_WP_125075606.1                   YFNLILGISLFV------FSIPQFLYFIVGFFLHIFKRK----------KVVYEC--KNHKFAI---LIPARNEEIVIKNLIDSLNKQNYPRELFEIFVIADNCTDK-----TKNVS---
14 Mbovigenitalium_51080_WP_051043965.1                    WFNISMLIFSLL------FLFLNILYFVVGFILRIFKNK----------KNSLMC--TMHKFAI---LIPARNESSVIANLIKSLKCQNYPSDLFDIYVIADNCTDN-----TKEIA---
15 Csphaeroides_BcsA_WP_011338158.1                        AVETFSISIFFLNGF---------------LSADPTDRPF--------PRPLQPE--ELPTVDILVPSYNEPAD--MLSVTLAAAKNMIYPARLRTVVLCDDGGTDQRCMSPDPELAQK-
16 Miowae_695_WP_004024575.1                               -TN----VLFFGLITSIFGFG-FLIYKI--FLKKHCIKVYSNECMEIINATKLPE--ILPKVLY---IYTCHND--LIESRVLQNKNQSY--KNFEIWVSDG-S--------------S-
17 Mgirerdii_VCU_M1_AIV03393.1                             -IN----VLFSSLAIPGYFLI-QSIWTT--LYKDYLWRKTKYEFLEIVHKTKIPE--KNNKVVF---VYTTHDD--FFESRFLQVTKQTY--KNIEYWISDG-S--------------E-
18 Mmoatsii_NCTC10158_V5E84_00300                          -QS----SLYAMSGITIINLI---IYQV--IYIKY-KKKLIWEYLEILSKAKVPS--KLPKVVY---VYTTHND--FMPSRLEQNIRQTY--RNIEYWISDG-S--------------S-
19 Mmoatsii_NCTC10158_V5E84_00290                          -IV----FLFVSIGISLFFIT---FLWF--LRKSY-IKKIVKDHLEITSRAKLPS--KLPKVVY---VYTTHND--FMPSRLEQNIRQTY--RNIEYWISDG-S--------------S-
20 Mmoatsii_NCTC10158_V5E84_00295                          -IV----FLFVSIGISLFFIT---FLWF--LRKSY-IKKIVKDHLEITSRAKLPS--KLPKVVY---VYTTHND--FMPSRLEQNIRQTY--RNIEYWISDG-S--------------S-
21 Mmiroungigenitalium_ES2806-GEN_WP_171111006.1           LLNIFTIDLFA-TFLMK--FF-LGLAFS--LSFRKTK---NKI-----RKIDVAS-MADAKIAL---LYPTKDD--FSPEAVLESINQSY--KNIHFFILDD-S--------------K-
22 Mzalophidermidis_CSL9942_WP_216505629.1                 LLNIFTIDLFA-TFLMR--FF-LGLAFF--LSFRKTK---NKI-----RKIDVTS-VADAKIAL---LYPTKDD--FSSEAVLESINQSY--KNIHFFILDD-S--------------K-
23 Mequigenitalium_T37_UUD36799.1                          -LASICQFYFWYIGIVD--FV-FWLFFW--INRSKNAKLIRKI-----AKTTVS--LDHARVLL---LYCTCDD--FNEEALIKSMQQNY--LNYKTVILDD-S--------------K-
24 Mbovigenitalium_51080_ENY68771.1                        -LNFLIILYFWFNGSYD--LF-SVLWYI--KNRKIIKNLHNKI-----NSIKIE--KNKHKIVL---AYCTCDD--FNENALLCSMKQDY--ENFRVVILDD-S--------------K-
25 Mprimatum_ATCC25948_WP_029513203.1                      -INIFIICYFWWLGIND--LM-QYIIGL--FEKRNNLKIINEI-----HSLPIS--LTDKKVIL---AYCTCND--FNEEALLTSSIQNY--LNYKVVILDD-S--------------T-
26 Mfelifaucium_ATCC43428_WP_051590866.1                   -LNAIFLMYFWLNGIKD--FL-YVCWYY--LFKKIIYKQYKKV-----LNTDVSN--VNDKVLL---VYCCCND--IVEDALSKCINQDY--KNYDVVILDD-S--------------T-
27 Mmucosicanis_1642_TQC51467.1                            -LNFIFLSYFWLNGVKD--TL-YVFFYY--IFKRKIYKQYKYV-----LRTDVSN--STDRVLL---LYPCCND--FVPNALEKSMQQNY--KYFDVVILDD-S--------------N-
28 Mleachii_PG50_ADR24180.1                                -INSIILSYFWLNSIKD--FV-YSLIFI--IRKKSILKKYDPI-----VKSVLTEEFKNKKVVF---LYCTCDD--FDPNALKKSMFQDH--INYEIVILDD-S--------------T-
29 Mcapricolumcapricolum_14232_KEZ20687.1                  -INSIILSYFWLNSIKD--FI-YSLIFI--IRKKSILKKYDPI-----VRSVLTEEFKNKKVVF---LYCTCND--FDPNALKKSMFQDY--INYEIVILDD-S--------------T-
30 Mcapricolumcapripneumoniae_M1601_AOQ21831.1             -INSIILSYFWLNSIKD--FI-YSLIFI--IRKKSILKKYDPI-----VKSVLTEEFKNKKVVF---LYCTCND--FDPSALKKSMFQDY--INYEIVILDD-S--------------T-
31 Magalactiae_14628_EIN15433.1                            -ANALIFCFFWLKSTKD--MV-FSLTFL--FSRKKLTKKFEPI-----INSSLSDEFKNKRVVL---LYCTCDD--FHEESLLSSMKQDY--LNYETVILDD-S--------------K-
32 Mbovis_F11436_V5E85_03235                               -VNALIFCFFWLKSTKN--MV-FSLVFL--FGRKKLAQKFEPI-----INSELTDEFKNKKVVL---LYCTCDD--FHEESLSKSMVQYW--NNYETVILDD-S--------------K-
33 Mmycoidescapri_PG3_EXU60752.1                           -INGTIFCLFWLKSTRD--LI-FSLFFI--IKKKSLLKLYHPI-----TKTKLRENFLSKKVVV---LYCTCDD--FSKDALLRSMNQDY--KNYEIVILDD-S--------------K-
34 Mmycoidescapri_PG3_EXU60168.1                           -INALIFCLFWLKSTKD--IV-FTFFFI--LKKKSLLKRYDYI-----INSELKDEFKNKKVIL---LYCTCDD--FDKDALKKSMNQNY--NNYEVVILDD-S--------------K-
35 Mferiruminatoris_14822_WFQ92340.1                       -INALIFCLFWLKSTKD--LV-FTFFFL--FKRKSLLKRYDYI-----INSELSEEFKNKKVFL---LYCTCDD--FDKEALKKSMKQNY--NNYEVVILDD-S--------------K-
36 Mpirum_ATCC25960_WP_162150122.1                         -INWIVINFITCIGIKD--FI-TMVTYF--FYIKNKPISKFYL-----KNEKLFD-KGQPKVLL---LYLTCDD--FDSNSLLKSIEQDY--DNFKIYILDD-S--------------K-
37 Mpirum_ATCC25960_WP_052663068.1                         -LNWLIINIGFCNGIKD--FV-ILLTYY--IYLKPKYKKNYFKQF---NHSKLIS-KLEPKVLL---LYTTCND--FDEQSLLQSINQNY--SNFCTYILDD-S--------------D-
38 Miowae_695_EGZ31427.1                                   -FNFIFVSSLYCSSIKN--FI-FTLAYL--FFYKRKNSRYVYTVIEN-KSLDIIL-KKNPMVFV---LYTTCND--FDSIALKKSLYQTY--QNCKFFILDD-S--------------N-
39 Mpenetrans_HF-2_BAC44425.1                              -FNFIFFSLLFCNNIKE--FI-QTLYYI--FIRKNIRAERVLRNKDYLLNKSKIN-NLNYVVFI---VYCTCND--FDEESITESLKQEY--KNCQFFILDD-S--------------N-
```

```
                                                         241          :          .          .          .     KAG .          3          DxD        .          .          .          :          . 360
 1 Mfermentans_PG18_BAH69904.1                               ---RNAGAI-VYEYSNSSERTKGYALKK---IFEFIEQD-------Y-----------GIQSFDGYHIFDADNIVDSLYFEKMNDAFLHYD----KKNVITSFR-------NTKNFGKSI
 2 Mbovigenitalium_51080_ENY68992.1                          ---LQAGADKVYERFNKQLIGPNFAVHE---TLLKIKDE-------F-----------G-D-FDSFSWFDADNIVDENWLSIMNDAFNDSK----KYDYFTSFR-------DTQNFEDNW
 3 Mcaviae_G122_WP_126118392.1                               ---REAGADKVYERFNKELIGPNFAIHE---TLLKIKDE-------F-----------G-T-FDSFSWFDADNIVEKEWLSKMNDAFNHPK----KYDYFTSFR-------DTQNFEDNW
 4 Melephantis_ATCC51980_WP_027334204.1                      ---RQGGADKVYERFNKELIGPNFAVHE---TLLKIKEE-------F-----------G-NIFDSFSWFDADNIVDKNWLSKMNDAFNHPK----KYDYFTSFR-------DTQNFEDNW
 5 Malvi_ATCC29626_WP_052663867.1                            AKKFPNRIT-VLERFNDKYRGANFAIQY---AIKYIRK--------------------FINIYDAYCYFDSDNIVDKHWLKEVVLQ--LNK----GYDVVTSYR-------NSINFKDNW
 6 Mferiruminatoris_14822_WFQ92977.1                         ---VKKNVV-VLQRFHKTLKGGNFAIQH---GLRYIRDN-------N-----------LLDKYDCFCSFDADNLLDQNWVYEVNKAYDFYS----DIQVVTTYR-------NSKNYADNW
 7 Mmycoidescapri_95010_CBW54549.1                           ---VKKEVV-VLERFHETLKGGNFAIQH---GLRYIRDN-------N-----------LLDKYDCFCSFDADNLLDENWVYEVNKAYDFYS----DIQVVTTYR-------NSKNYGDNW
 8 Mmycoidescapri_95010_CBW53832.1                           ---IEKGIT-VLERFHSTLKGGNFAIRH---GWRYIRDN-------N-----------LLDKYDCFCSFDADNLLDENWVYEVNKTFDFYN----DIEVVTTYR-------NSKNYADNW
 9 Mmycoidesmycoides_PG1_CAE76760.1                          ---IEKGIT-VLERFHNTLKGGNFAIRH---GWRYIRDN-------N-----------LLDKYDCFCTFDADNLLDENWVYEVNKTFDFYE----DIEVVTTYR-------NSKNYGDNW
10 Malligatoris_A21JP2_EFF41433.1                            ---KEAGIN-VLERFNNVQKGGNFAIQH---ALRYIRDN-------N-----------WLENYDCFCSFDADNLLDKNWVSEINKAYDYYG----S-DVVTSYR-------NSKNYADNW
11 Mcrocodyli_MP145_ADE19752.1                               ---ISKGVT-VLERFNDIQKGGNFAIQH---ALRYIRDN-------N-----------LLDNYDCFCSFDADNLLDKNWVFEVNKAFDFYG----S-EVVTTYR-------NSKNYADNW
12 Mgallinarum_DSM19816_WP_027332911.1                       ---KEYNFN-IYLRQNFEIKGKGQLVNE---MINFAN---------Q-----------NNLLTDAFIIMDADNLLDENFTDAMNNA--YNQ----GYEVAVGYR-------NNKNWNSSW
13 Mcalifornicum_HAZ106_1_WP_125075606.1                     ---LEAKAN-VIEIFNTELIGKSYALDY---AIKKISNMSQN----------------MEKPYDAYIVFDADNVVDKDFLKYMNIE--YSK----GYKVVTSYR-------TAKNFNSSL
14 Mbovigenitalium_51080_WP_051043965.1                      ---DQQGAI-VIERNDAQNIGKGYTLDY---AIKHINQLRRGGGQLS-----------NSLEYDAFIVFDADNVVDKDFLHYINIE--YSK----GYKIITSYR-------MPKNYNSSW
15 Csphaeroides_BcsA_WP_011338158.1                          ---AQERRR----------ELQQLCREL---GVVYSTRERNEHAKAGNMSAALERL-----KGELVVVFDADHVPSRDFLARTVGYFVEDP----DLFLVQTPHFFI----NPDPIQRNL
16 Miowae_695_WP_004024575.1                                 ---NQEWKN----------KIEKFCKDN---NINLFQLPKPSVNKADNLNQFLN---FYKDDFDYLLIGDADEVFHKNFVEYAIKIFYSNK--IKNLAYVTPLNINY----RSKGIYPNT
17 Mgirerdii_VCU_M1_AIV03393.1                               ---KKHNIK----------RVEEFAKKH---NINYFSLGRPSINKGDNLNTFIL---KSGAKFDYIVIMDADVHVDINFVETSLKLFNSENNKSKKLGYISPCVFNY----GSKSLFSNW
18 Mmoatsii_NCTC10158_V5E84_00300                            ---NPDNSK----------LVEEFAKKH---KINLFKMNQPSKDKSDNLNAFLNHLYSLNHDFEYIVTSDSDVAFDKKLVECNLRYFYTEN--QIRLGYVSSMIQDY----KTKNFFSNM
19 Mmoatsii_NCTC10158_V5E84_00290                            ---NPDNSK----------LVEEFAKKH---KINLFKMNQPSKDKSDNLNAFLNHLYSLNHDFEYIVTSDSDVAFDKNFVEVGIRYFYSEK--INRLGYVNSLLIDY----NSNNFYNNG
20 Mmoatsii_NCTC10158_V5E84_00295                            ---NPDNSK----------LVEEFAKKH---KINLFKMNQPSKDKSDNLNAFLNHLYSLNHDFEYIVTSDSDVAFDKNFVEVGIRYFYSEK--INRLGYVNSLLIDY----NSNNFYNNG
21 Mmiroungigenitalium_ES2806-GEN_WP_171111006.1             ---KPKYIS----------MIDSFIQQNQHLNITVIRRKIKKGFKAGNINNFLLSY---KNEFDYYVWLDSDEKLCFDFVERCLQYFYYYS----NLGIVQGNHKSD----SPQTQFQNL
22 Mzalophidermidis_CSL9942_WP_216505629.1                   ---KSKYIS----------MIDSFIQQNQHLNITVIRREIKKGFKAGNINNFLSLY---KNQFDYYVWLDSDEKLCFDFVERCLQYFYYYS----NLGIVQGNHKSD----TPQTQFQNL
23 Mequigenitalium_T37_UUD36799.1                            ---TQKFKD----------KIDLFKIKYP--NVKVIRRETNIGYKAANINNYLKTR----SDYDYFVLLDSDEIIPSNFITSCLKYFNYFT----NVGVVQANHKGK--G--GVNDFQKL
24 Mbovigenitalium_51080_ENY68771.1                          ---SQEYIE----------KIDNFAKKYK--NVSVVRRTKKVGFKAGNINNYFLNN----KDWDYFVILDSDEVIPSNFITQALKYFYHFK----NIGVLQANHRGI--N--ALNDFQKT
25 Mprimatum_ATCC25948_WP_029513203.1                        ---NKEYID----------KINEFVKKYP--NIELVRRKTKEGFKAGNINNYFLNK----SDYDYLVILDSDEIIPTNFIIESLKYFKYYS----NVGIVQANHKGR--STGITNNFQKW
26 Mfelifaucium_ATCC43428_WP_051590866.1                     ---KPEEIQ----------KVNNFAKTHSNINIKIVRRSNRKGFKAGNLNHYLMSDECKSKNYKYYVILDSDEIIPRNFIRSALKHFYAYE----NIGIVQANHIAT----NNKNFFMDL
27 Mmucosicanis_1642_TQC51467.1                              ---DESEKK----------IVDDFALKH---NIKVVRRKDRVGFKAGNLNNYLKSQECLKKQYDYYVILDSDEIIPNNFIELALKYFYTFS----NVGIVQANHIST----NNSNFFMGL
28 Mleachii_PG50_ADR24180.1                                  ---KLEYIS----------KIDEFSKKH---NIKVIRRKNREGFKAGNLNNYLKNN-----DYDYFVVLDSDEILPNNFISESLKYFQYDQ----NIGVVQAVHLAA----KPNNFYQYL
29 Mcapricolumcapricolum_14232_KEZ20687.1                    ---KLEYIS----------KIDEFSKKH---NIKVIRRKNREGFKAGNLNNYLKNN-----DYDYFVVLDSDEILPNNFISESLKYFQYDQ----NIGVVQAVHLAA----KPNNFYQYL
30 Mcapricolumcapripneumoniae_M1601_AOQ21831.1               ---KLEYIS----------KIDEFSKKH---NIKVIRRKNREGFKAGNLNNYLKNN-----DYDYFVVLDSDEILPNNFISESLKYFQYDQ----NIGVVQAVHLAA----KPNNFYQYL
31 Magalactiae_14628_EIN15433.1                              ---SEEYIK----------IVDTFAKEH---GLRVVRRENKEGFKAGNLNNYLKNN----DDYDYFVVLDSDEVIPNDFITQSLKYFQFNN----KIGALQAYHINKTKVNGGGNLFQYL
32 Mbovis_F11436_V5E85_03235                                 ---SNEYIE----------KVNEFANKH---NLKVVRRENKEGFKAGNLNNFLKHN----DDFDYFVVLDSDEVIPADFISQSLKYFQFDE----KIGALQAYHLNK----KGKNLFQYL
33 Mmycoidescapri_PG3_EXU60752.1                             ---KLEQIK----------EIDEFSNQY---NIKVIRRTNRIGYKAGNLNNYLKNN----PDYDYFVVLDSDEIIPNNFITESLKYFQFDE----KIGAVQAYHAAS----KGKNLFQYL
34 Mmycoidescapri_PG3_EXU60168.1                             ---KPEYIK----------RVDEFSKKY---NIKVVRRNDRVGYKAGNLNNYLKNN----PDYDYFVVLDSDEIIPNNFITDSLKYFQTNK----KIGAIQAYHLIS----KGKNLFQEA
35 Mferiruminatoris_14822_WFQ92340.1                         ---KEEYIK----------QVDDFSKEY---NIRVVRRENRIGFKAGNLNNFLKNN----TDYDYFVVLDSDEIIPNNFIIESLKYFQSNP----KIGALQAFHLIS----EGKNFFQQA
36 Mpirum_ATCC25960_WP_162150122.1                           ---KDTFKN----------EINDFCAKYQ--NINLIRRNNISGFKAGNINNFLMNT---KEFFDYFVILDSDEIIPKNFIKECLLYFTN------DIGIIQANHKAT----RTSNFFDKL
37 Mpirum_ATCC25960_WP_052663068.1                           ---QEFYKN----------KIDNFINKNK--NINLVRRKDKKGFKAGNINNFLMNT---KEEFDYFVLLDSDEIIPNNFIKNCLPYFEN------NVGIVQANHKAT----RFSNLFDKL
38 Miowae_695_EGZ31427.1                                     ---DSKYKD----------EIDKFAYEY---NIEVIRRKNRKGFKAGNINNFLMNR---N-DYDYFVLLDSDEIIPKDYILKVLPYFS-NK----KVGIVQCNNKCN----RQNNYFDYL
39 Mpenetrans_HF-2_BAC44425.1                                ---KKEYKD----------QVNKFARKYE--NVKVIRRKNNKGFKAGNINNFLLKR---K-DYDFFIILDADEIIPSDFVGKSLMYFEENK----ELGILQAKNLCT----RQTNWFDYI
```

```
                                                   361        .         .         .         4         .        .ED        .         .         :       QxxRW         .        . 480
 1 Mfermentans_PG18_BAH69904.1                     QTINYG-LMFMFHTTIESNARMKFKMSSKILGSGFLISNE-MVKD---GW---N-ITIPSDDSDFTVEQISEGKNVKYCDEAMFYDEQPTKFKDMWRQRLRWVFGNRIVW--------RK
 2 Mbovigenitalium_51080_ENY68992.1                ISSAYS-IEFYRLVSQVATTRSAFRNPHMVGGTGFMVRWS-LLEK-NNYW---GKYQSMVHDTEFSFDALVNGYKGVYVDQAKFYDLQPTDWNISWKQRTRWTVGNYKLG--------NL
 3 Mcaviae_G122_WP_126118392.1                     ISSSYS-IEFYRLVSQIATVRSVFKNPHMVGGTGFMVRWE-ILEK-NDYW---GKYKSMVHDTEFSFDALSNNYKGIYVDGARFYDLQPTKMSVSWKQRTRWTVGNLKLG--------HT
 4 Melephantis_ATCC51980_WP_027334204.1            ISSSYS-IEFYRLVSQIATARSFFKNPHMVGGTGFMVRWS-LLEK-NNYW---GKYTSMVHDTEFSYYALLNNYKGIYVDEAHFYDLQPTNWNISWKQRTRWTVGNYKLN--------KS
 5 Malvi_ATCC29626_WP_052663867.1                  ISASYA-IQFIKESNYINASRNRFNMTSWINGTGFCFSEK-VLQM-SNDW---D-FNTLSHDIEFTQFLSINNIKCGYANDAIIYDEQPIKFKDSYAQRLRWSKGFFQVF--------KL
 6 Mferiruminatoris_14822_WFQ92977.1               ISSAYS-IQFLKESDVINKGRATLNHTSYINGTGFSFTKE-ILEK-TNWW---D-FNSLSHDIEFTQWLMLNNIKTGYTENACFYDEQPIDFKSSWKQRMRWSVGFKQVW--------NI
 7 Mmycoidescapri_95010_CBW54549.1                 ISSAYA-IQFLKESDIINKGRATLNHSSYINGTGFSFTKK-ILEQ-TNWW---D-FNSLSHDIEFTQWLMLNKIKTGYTEDACFYDEQPIDFKSSWQQRMRWSVGFKQVW--------QI
 8 Mmycoidescapri_95010_CBW53832.1                 ISSACS-IQFLKESDIINKGRATLNHSSYINGTGFSFTKK-ILEQ-TNWW---D-FNSLSHDIEFTQWLMLNKIKTGYAPNACFYDEQPIDFKNSWKQRMRWCVGFKQVW--------KI
 9 Mmycoidesmycoides_PG1_CAE76760.1                ISSAYS-IQFLKESDIINKGRATLNHSSYINGTGFCITKK-IFEQ-TNWW---D-FNSLSHDIEFTQWLMLNKIKTGYTEDACFYDEQPIDFKSSWKQRMRWCVGFKQVW--------KI
10 Malligatoris_A21JP2_EFF41433.1                  ISSAYS-IQFLKESTVHNKGRSSKQHSAYINGTGFSFTKE-VLEQ-TKWW---D-FNSLSHDIEFTQWILLNKKKCNYSEDAIFYDEQPIKFKDSWKQRMRWSVGFKQVW--------NL
11 Mcrocodyli_MP145_ADE19752.1                     ISSAYS-IQFIKESSVINKSRNYKRYSAYVNGTGFSFTKK-ILEQ-TNWW---D-FNSLSHDIEFTQWLLLNNVNCAYSENAIFYDEQPIRFKDSWKQRMRWCVGFKQVW--------NI
12 Mgallinarum_DSM19816_WP_027332911.1             VSACSG-LTFSRFSHLENLKRTKNGTTIQLSGTGFYISKK-QIEK-FGGW---N-FFTLTEDLELTINMALDNTKCAYVSDAIFYDEQPIKLNDANKQRLRWIKGHNQLF--------RK
13 Mcalifornicum_HAZ106_1_WP_125075606.1           IAGISG-LLFVRESIFLQRPRKTLKLNCSISGTGFLVDAK-LLN---NEW---K-YNLLTEDIELSTDMVIKGIKCGYAKDAITYDEQPEDIKTFYKQRIRWAKGFLQVF--------GK
14 Mbovigenitalium_51080_WP_051043965.1            VAAISG-LMYIRESVFVNRPKKALNISCSISGTGFLVDSN-ILK---DGW---K-YHLLTEDIQLSSDLVSKGQKIGYAKDAITYDEQPTDLKTFFRQRTRWAKGFLQVF--------RK
15 Csphaeroides_BcsA_WP_011338158.1                ALGDRCPPENEMFYGKIHRGLDRWGG-AFFCGSAAVLRRR-ALDE-AGGF---A-GETITEDAETALEIHSRGWKSLYIDRAMIAGLQPETFASFIQQRGRWATGMMQMLLLKNPLFRRG
16 Miowae_695_WP_004024575.1                       T--RII-ETTAFYWSLFTKNFNLANV-SPLAGQSCLISKKSLILCYKNGK---F-ENVNLEDWYLEARMVEEGNFGIMLPNAPCYFEPDVNVLVHFNRIMRIEDWRVRWW--------KI
17 Mgirerdii_VCU_M1_AIV03393.1                     G--RNF-FGVFQCLAEQIRFFSPFDR-TELFGACAIIKKE-MLVDINKNKKPLF-SEGCLEDVYTSWFGCKQGWNGVINVLSNCSEMFDRTIFALHKRLLRIYDWTVKYT--------KE
18 Mmoatsii_NCTC10158_V5E84_00300                  L--RFV-ENLTLFKKDINKVLRTNYS-PNLYSACCLLKKE-YLDDMNN-F-----PPGFFEDGYLELNGSKKYWYGLISPLTLSLQKFDENIKKYKTRQLRIIDWIIKYE--------QS
19 Mmoatsii_NCTC10158_V5E84_00290                  M--AGW-ETVPNFFRTIFNNLDMSSP-AHIVGASSIVKRT-FLEEVNKGY-----FFNIFEDWYTRMNGIKIMWTGLHTPLVTSQQSMEDNVFPTFKRYMRYFSWKAAFF--------KK
20 Mmoatsii_NCTC10158_V5E84_00295                  M--AGW-ETVPGFLMPILENLDNLRT-PHLYGACSITKVS-FLKDINNGY-----FFDIYEDWYTRMNGIKKMWKGLYSPLVVSQQSMEDNVFPTLKRYMRYFSWKAAFF--------KK
21 Mmiroungigenitalium_ES2806-GEN_WP_171111006.1   L--SRC---IPWKLPIINEYYNFIGH-SFLYGHGAMISND-ALSAMDFKI-----PEIVIEDIATSIVAKSNKYQTIFASELVNTEEFPISFNAFYVRQLKFVGGDHDLWFRV---LIRK
22 Mzalophidermidis_CSL9942_WP_216505629.1         L--SRC---IPWKLPIINEYYNFIGH-SFLYGHGAMISNE-TLSTMDFKI-----PEIVIEDIATSIVAKSNKFQTIFASELVNTEEFPVSFNAFRVRQLKFVSGDHDLWFRV---FIRK
23 Mequigenitalium_T37_UUD36799.1                  F--SYF---ILPSLSINLVTKNEYGI-VSLFGHGAMVSRQ-CYESTKNGF-----PELISEDNAFTIQALNNNFFVYFAPNIICEEDFPVNYFAFKKRQTKFINGNLQFN--------SL
24 Mbovigenitalium_51080_ENY68771.1                F--DTS---IISGLTANLTAKNHTSI-VTLFGHGAMISKE-CYESA-GGF-----PHLVSEDNAFSATILAKGYYVVYFAPDIICGEEFPTNYLAFKKRQSKFVSGDMQLF--------SK
25 Mprimatum_ATCC25948_WP_029513203.1              F--SVS---ILPSLFKSLWPRNKNGL-VNCFGHGAMISKE-CFDKV-GGF-----PQVVSEDNAFTLKMLYENIFVVFAPNIICQEDFPINLSAFIKRQGKFINGNIEMS--------KM
26 Mfelifaucium_ATCC43428_WP_051590866.1           F--HRG---VNSHWPVYQTMKHKFGF-STMLGHGAMIKAE-CYDKLDNGF-----PPMVAEDLCISIELRSKGYLVSFAPEIICQEEYPVDYIAFKKRHSKWTQGNLEFI--------KK
27 Mmucosicanis_1642_TQC51467.1                    F--HVG---VNSHWPVYQSMKNYYGF-STMLGHGAMIKAE-CYNMLNEGF-----PNLVAEDLCISIELRSLGYYVVFAPNIICQEEYPIDYIAFKKRHSKWTQGNLEFI--------KK
28 Mleachii_PG50_ADR24180.1                        L--GLS---IDSNSLVTQVMKNFYGS-NSLLGHGMMISKE-CYKK-TNGF-----PHIVAEDIAFAIEVKDAGYKIAYAFNIFCKEEFPNDYISLKKRQCKWTQGNVEYM--------KK
29 Mcapricolumcapricolum_14232_KEZ20687.1          L--GLS---IDSNSLVTQVMKNFYGS-NSLLGHGMMISKE-CYKK-TNGF-----PHIVAEDIAFAIEVKDAGYKIAYAFNIFCKEEFPNDYISLKKRQCKWTQGNVEYM--------KK
30 Mcapricolumcapripneumoniae_M1601_AOQ21831.1     L--GLS---IDSNSLVIQVMKNFYGS-NSLLGHGMMISKE-CYKK-TNGF-----PHIVAEDIAFAIEVKDAGYKIAYAFNIFCKEEFPNDYISLKKRQCKWTQGNVEYM--------KK
31 Magalactiae_14628_EIN15433.1                    L--SVS---SNVSSLDNHYMRQLYGE-NSLLGHGMIISKE-VYRQ-TNGF-----PHILVEDTSMSAEIKALGYEVVYAPNIVCYEDFPNDYIALKKRQCRWTAGNVQYI--------KK
32 Mbovis_F11436_V5E85_03235                       M--SIS---ANAQSLDHHYMRQLYGE-NSLLGHGMIIRKD-VYRQ-TNGF-----PQILVEDTSMSAEIKSIGYDIVYAPNIVCYEDFPNDYIALKKRQCRWTAGNVQYI--------KK
33 Mmycoidescapri_PG3_EXU60752.1                   L--GDS---NNNTALSFHIMRDIFGE-TSLLGHGMILSKQ-AYEK-TGGF-----PHLLVEDTSMSAELKKQGFKIAYASNILCYEDFPTNYIALKKRQSRWTAGNVQYI--------RK
34 Mmycoidescapri_PG3_EXU60168.1                   V--GNS---SNIASLSIHVLRNFYGE-TSLAGHGMMLSKE-VYEK-TGGF-----PHLLVEDTSMSAEIKKNNYEIVYAPNIVCYEEFPINYIALKKRQGRWTAGNVQYI--------KK
35 Mferiruminatoris_14822_WFQ92340.1               I--GNS---SNVSSLSIHVLRNLYGE-TSLAGHGMMLSKE-VYEK-TGGF-----PHLLVEDTSISAEVKKNNYEIVYAPNIVCYEEFPVDYIALKKRQGRWTAGNVQYI--------KK
36 Mpirum_ATCC25960_WP_162150122.1                 A--SNH---VLPYWTISMSFKNLYGT-TPFYGHGAMISRN-CYFD-CNGF-----PEIVTEDIGFLIKSLNKGYKSLFLPHVICEEKFPIDYLAYKKYLNKITQGHIELI--------KK
37 Mpirum_ATCC25960_WP_052663068.1                 G--SKG---VLPAWTTFISFKNVYGS-VTLNGHGAMIDRK-CYYD-SMGF-----PEIVAEDLGFAVNAINKKYKIVFAYDVICEEKFPVDFISFKKRNIKWTQGNFEFI--------KK
38 Miowae_695_EGZ31427.1                           G--SYV---HNTQWNTEYVVRHSLGV-VNCCGHGATISKK-MYLD-VGGF-----PELILEDWALTLNGLKFGYETVYAPEIVCWEEFPTNYLAFKKRNYRWTQGGVQCF--------SK
39 Mpenetrans_HF-2_BAC44425.1                      G--SYI---HNYQWQNQYNVRNSIGM-LNLCGHGAMIKRE-CYEK-SGGF-----PEILLEDWALTFQALKNNFLTAYANNIVCYEKFPSDYISFKKRQFRWTMGGVECF--------KK
```

```
                                                481         .         5         .         .         .         :         .         .         .         .         6 600
 1 Mfermentans_PG18_BAH69904.1                      RFKSLIKN---IFSSKKEDDKKISKSSSIDILFMISP-ISFIGFLILL----LNILLVS-LG--PI--------F--------------GANATQMWIDW-AISFSISHAIIYLILFVIG
 2 Mbovigenitalium_51080_ENY68992.1                 INKTLFKN---LFFKFEAPQKKLN---YLDYIAMLTP-SWLFFFTLLI----INTTITA-LN--LV------L-L--------------ANNIDNIGVYLGALSLPIMFIYYYLITFIMG
 3 Mcaviae_G122_WP_126118392.1                      IWGKIFKN---LFFKFEAPQKKLN---YLDYIAMLSP-AWIWFVVLFI----FNTTLTV-LN--GV------L-L--------------GFGQVDFWKFLGVLSLPLMFVWYYLSAWFMG
 4 Melephantis_ATCC51980_WP_027334204.1             IKWSIFKN---LFFKIEWPQKKLN---YLDFIAMLFP-ASFWFILLLI----INIASSI-TN--GL------IYF--------------GFNLHNTYYLLGFLSIFSMFLFYYLTSFFMG
 5 Malvi_ATCC29626_WP_052663867.1                   YSFKEVKQ---LFLFWKKE--NKNIKSIYANLALIFP-Q----VFLFL----TSLFLYV-LI--SILLS--CVS--N---------NELCKLIIIPWMFW-IYTPIAIFSGLYLTFLVFA
 6 Mferiruminatoris_14822_WFQ92977.1                YKSRILKQ---MFSL------KANKVKIWANFTMISP-V----VLTLV----INLLFWL-IT--TLLMV--SNYEVNYVHNRFIQVAQVQNNFYHLLVYA-LTTPFVIHLIIFVNYLLWA
 7 Mmycoidescapri_95010_CBW54549.1                  YKSKMIKN---MFSS------KVNKIKLWVNFTMISP-V----VITLV----INLLFWL-IT--SGLMI--SNYIINFSFNQAITDIQMHKQLFNLIIYI-ISTPLVIHLIIFINYFLWG
 8 Mmycoidescapri_95010_CBW53832.1                  YRSEMIKN---MFTF------KINKIKLWVNFTMIFP-A----VITLL----INLLFWL-IT--SGLMI--SNYIVNYLNSSLVIIEQANNYLRDLIIYC-TTTPIVIFGIIFINYLLWG
 9 Mmycoidesmycoides_PG1_CAE76760.1                 YRSKMIKN---MFTF------KVNKIKLWVNFTMIFP-A----VITLM----INLLFYL-IT--SGLMV--SNYIVNYLNSSLVMVEQVNNYLRDLIIYF-TTTPLIILGVIFINYLLWG
10 Malligatoris_A21JP2_EFF41433.1                   YKKSLVKM---MFKP------KLKHFPVMMNILTLFP-G----IYTFI----INILFYV-IT--SVLMF--LNYYYNYLTDTS---AFAAFNLTQVIIYI-TTTPFFILLTIYLNLFIQG
11 Mcrocodyli_MP145_ADE19752.1                      YKNRLNTA---LVKK------NINKPSLITNYIMISP-A----IYTLI----INISFYL-IT--SISMV--LMYFINYTLDPV---TYVDYNLFGLILYI-LLTPFVIIITIYLNLFVQA
12 Mgallinarum_DSM19816_WP_027332911.1              YRKSLNEA---LFKKSQSN----------KVGCFELK-FQAVSFASFI----LSIIFII-AT--ISLGI--WTFYISHLN---------QK----ILITI-ISNLIAILLGLIIYYFSIV
13 Mcalifornicum_HAZ106_1_WP_125075606.1            YSCGLLSK---FIRSLSAI----------DFFTFIFP-LTGLIVLGLL----VNL----------TFMT--VGYISNDIN-------LGNA----GLFEI-IKT---IVIIYLISFF-ML
14 Mbovigenitalium_51080_WP_051043965.1             YSGKLFLK---SFTSISAF----------DIFSFIFP-TSLIFILSFL----VNL----------SFAI--FGYSTSNIA-------LGNY----AIINL-TIG---ISLTLLTPFV-FG
15 Csphaeroides_BcsA_WP_011338158.1                 LGIAQRLCYLNSMSFWFFPLVRMM--------FLVAPLIYLFFGIEIF--------------------V--------------------ATFEEVLAYM-P--------GYLAVSFLVQ
16 Miowae_695_WP_004024575.1                        NNKKIINNFNERFVNWYKNY--IG--------VLFRP-I-IIF--------------SSIGLLGLVIWLISNYYN--------------YAFKNNVLFWI-SLG---TGFGFGLITLLLN
17 Mgirerdii_VCU_M1_AIV03393.1                      NKF---KNYQERYENREMID--FL--------IVILP-IILIFVILVV---LPAV---------SSLFL--IFFS---------------ETISNIYLWL-PQL---VFLSVITISFLFY
18 Mmoatsii_NCTC10158_V5E84_00300                   EAF---VNINNKFKSTYNEY----------------------FINLFS---GPAILLSLILLPLFILFL--VWFW--------------PIFITTLYYWI-ILG---MWLISGWI-----
19 Mmoatsii_NCTC10158_V5E84_00290                   NTF---YRFNDEYSKNFWSE--FN--------PVMFY-IFKIFLNLFV---FTSFIFLLIHSVKSTIFL--FFLI--------------PLFLYIILFWS-NSF---------------
20 Mmoatsii_NCTC10158_V5E84_00295                   NTF---YRFNDEYSKSFWNE--LN--------SIIFY-VFKIFINLFI---LSSCILLTMISIRSIMFF--VFLI--------------PIFLYKFLFWS-NSF---------------
21 Mmiroungigenitalium_ES2806-GEN_WP_171111006.1    IGRHNNI--------FSMFQILSN--------ISLSNILFLVFNLLILIFQIVSNVFSVVSN-IDLNLY--FGFE--------------Y----NICIIL-LII---GSFIATLIPVFIH
22 Mzalophidermidis_CSL9942_WP_216505629.1          IGRHNNI--------FSMFQILSN--------ISLSNVLFLVFNLLILIFQIISNVFSVASN-IDLNFY--FGFK--------------Y----NIFIIL-LIT---GSFIASLIPVFIH
23 Mequigenitalium_T37_UUD36799.1                   I-LKKEY--NIKAPAWQRWNFLTQ--------IGLVY-LTIILGFLFL------------IS-TTVLVC--LKSN--------------NYK-EMIIILY-F------SIFLMVAPWLKE
24 Mbovigenitalium_51080_ENY68771.1                 SVIQNIF--KSKISWFLKFDLLNR--------FSMIY-LLVFFGFLFT------------LF-NISIYF--LNGD--------------LFF-KSKYFTI-V------SCLFIIAPWVKE
25 Mprimatum_ATCC25948_WP_029513203.1               I--VKKR--KGNYPKWLKFELFSH--------VSMIY-LTIIFGFIFQ------------FN-NVLIFT--FNRD--------------FIN-HNIYIYL-I------SLFFIFIPWIKE
26 Mfelifaucium_ATCC43428_WP_051590866.1            FTKNISK---AKMNWFEKMDIFLF--------TYNLP-LTALFSFYIF------------IN-IIVLPL--IGVN--------------LGSVYPAWFLI-P------TIIFFFSPMIND
27 Mmucosicanis_1642_TQC51467.1                     FTGKIIK---SKMSWFEKLDIFLF--------TYNLP-LTAIFAFNIF------------IN-IMILPI--IGVS--------------ITKIFPIWLLI-I------STIFFFAPMLND
28 Mleachii_PG50_ADR24180.1                         YSKNIKK---SSYKWYEKLDIKLS--------HYSLP-IVPMFSLILI------------IN-TSLLGF--LEYR--------------IDP-YRIALLS-M------SLLFLISPVIPN
29 Mcapricolumcapricolum_14232_KEZ20687.1           YSKDIKK---SSYKWYEKLDIKLS--------HYSLP-IIPVLSLILI------------IN-ASLLGF--LEYR--------------IDP-YRIALLS-M------SLLFLICPIIPN
30 Mcapricolumcapripneumoniae_M1601_AOQ21831.1      YSKDIKK---SSYKWYEKLDIKLS--------HYSLP-IIPVLSLILI------------IN-ASLLGF--LEYR--------------IDP-YRIALLS-M------SLLFLISPVIPN
31 Magalactiae_14628_EIN15433.1                     YAKKNRK---SNYRWFERIDLVLN--------HYSLP-LIPVFAVIFL------------IN-FLIIGF--LGFR--------------ISP-RELVFVI-I------WISFLIGTLLLS
32 Mbovis_F11436_V5E85_03235                        YAKKNRK---SKYRWFERIDLILN--------HYSLP-LIPVFALLFL------------IN-FLIIGF--LGFT--------------VST-KELTFAI-I------WMVFLLGSLLLS
33 Mmycoidescapri_PG3_EXU60752.1                    YGKQNRK---IDYKWFERIDLLLN--------HYSLP-LVPIFGFIFL------------IN-FIILGF--IDFQ--------------ARF-YETAFII-I------WILFLLSPILVN
34 Mmycoidescapri_PG3_EXU60168.1                    YAKQNRR---TKYKWFERVDLRIN--------HYSLP-IVPVISLVFL------------LN-FIILGF--LKFN--------------ITT-NDIAFVI-I------WCAFLFLPLFVN
35 Mferiruminatoris_14822_WFQ92340.1                YAKVNRK---INYKWFEKIDLRLN--------HYSLP-IIPIISLIFL------------IN-FIILGF--LKFN--------------ITT-NDIAFVI-I------WCIFLFLPLFIS
36 Mpirum_ATCC25960_WP_162150122.1                  LGTEIFV----KKNFFMKLSLFFD--------VTSIF-INLYSILLLI------------IS-FLILGP--LNFQ--------------YK--FQYIIWI-F------LIFFNFIYLISD
37 Mpirum_ATCC25960_WP_052663068.1                  FGLKLII---NKTNFFKKLDLFLS--------STSIF-LTVFCFFTII------------IN-FSILYP--LGFY--------------YK--YSYFLWI-F------IVFFFLIPLLND
38 Miowae_695_EGZ31427.1                            IGWKLLF--FNSCPFFRRLDLLLT--------QTNYF-ISIGSLIIVL------------ST-LSILAP--MGFR--------------FR--YENWYVA-V------TLFFAIIPLLNC
39 Mpenetrans_HF-2_BAC44425.1                       IGWKIFF--F-KAPFFKKIDIFLN--------QASFF-ITIFSLLSLV------------IN-ISILYP--LNFS--------------FS--YFDWYIL-L------SVILTSIPLINL
```

```
                                             601                                                                                                                  720
                                                      .        .        .        .        :      FxVTxK     .        .        .       7        .        .
 1 Mfermentans_PG18_BAH69904.1                IITYARGRKRVQRVPWW--KMIISLFIWPLFLALYF--------PLQIVAIFKNINKFN-WTQIEHNKNI--------------------------------------------------
 2 Mbovigenitalium_51080_ENY68992.1           LCVIIRQARKIKRFSSW--NKFKALITYPIFIFLNI--------PISMYASKNKNMKFV-NTQKQRSLNTKI------------------------------------------------
 3 Mcaviae_G122_WP_126118392.1                LAVIIRQCKKMKRISGW--SKFKALFGYPIFLFLNV--------PISIYAQKNINMKFV-NTKKERSTKTMI------------------------------------------------
 4 Melephantis_ATCC51980_WP_027334204.1       LAVIIRQSKKMKQISFW--NKIKSLFFYPIFIFLNI--------PISFYAQKNINMKFV-NTKKERTKKF--------------------------------------------------
 5 Malvi_ATCC29626_WP_052663867.1             LAVVIQEKKRID-CKLY--KGIIYAFMYPFFMFTYV--------PISIICIFSK--KVS-VNPIKRKTRI--------------------------------------------------
 6 Mferiruminatoris_14822_WFQ92977.1          IVVVIRNRKSIQ-ATKW--QKFKSIFTYPLFMLTYI--------PISFLALFRS--TYS-TTPIARKSEVVLKTKPNKTSNK--------------------------------------
 7 Mmycoidescapri_95010_CBW54549.1            FIIVARNKKSIQ-ATNW--QKFKSVFTYPFFMLTYI--------PISFLALFKS--TYS-TTPICRKAELDLKTKQAKKLDK--------------------------------------
 8 Mmycoidescapri_95010_CBW53832.1            FIIVARNKKSIQ-ATSW--QKFKSIFTYPFFMLTYI--------PISFLALFKS--TYS-TTPIARKAQLVQN-----------------------------------------------
 9 Mmycoidesmycoides_PG1_CAE76760.1           FIVVTRNKKSIQ-ATSW--QKFKSIFTYLFFMLTYI--------PTSFLALFKS--TYS-TIPIPRKEELVQS-----------------------------------------------
10 Malligatoris_A21JP2_EFF41433.1             IIILKRNSKRIN-ATKW--QKCKSLFTYPLFMITYI--------PITFIALFKK--SYS-TAPIARKQ----------------------------------------------------
11 Mcrocodyli_MP145_ADE19752.1                IIIVKRNWKRIN-AKKS--KKIISIFTYPLFMLSYI--------PISFIALFKK--TYS-TTPIQRKENQD-------------------------------------------------
12 Mgallinarum_DSM19816_WP_027332911.1        IYTALSIYADPR-VAMTTKMKWKACFMQPIYLFWYI--------PLFIKALCKK--EVQ-WTKIDHSINIDLNDLQNK------------------------------------------
13 Mcalifornicum_HAZ106_1_WP_125075606.1      FTTTILEWNKLK-APFY--KKILYIFISPLAVFIYA--------IIFIIALFKR--KVI-WTQIKHTSNQNIDQM---------------------------------------------
14 Mbovigenitalium_51080_WP_051043965.1       LITTLTEYKRIK-ASFI--KKIWYSILFPIAFFIYL--------AAFLTALLKK--KVV-WKPIKHSDTKDIVQM---------------------------------------------
15 Csphaeroides_BcsA_WP_011338158.1           NALFA----RQR----W----------PLV-SEVYEVAQAPYLARAIVTTLLRPSARFAVTAKDETLSENY--ISPI---YRPLLFTFL--LCLSGVLATLV-----------------R
16 Miowae_695_WP_004024575.1                  SILIG------K----INYNFWDYILFPFIFVLWFLAA-NIKITIHWFRSLFLGKYSQFGGSGNSRFFKSKSK---TIKWWISFLILS----ITITIFNVCFF-------ILLNWSMY-K
17 Mgirerdii_VCU_M1_AIV03393.1                YLYFN-HYSQ--------KGIVYRICSLITFFLIIIITLLPEYVKHFINSYFFSKYSDFTPSIKTDKQAQNEKKLI----YLLRFKILLVIATSILIFLLVFFGIKSGMFINPKQNILFC
18 Mmoatsii_NCTC10158_V5E84_00300             ISAIA-NYDSAK----IMGKANAF-FFPLF-QFLLFMSYWPSLVKHWFKAFVLKKYSTFMPSRARNYKNKKQSKFQPIKFDLLIMLISLTI-LGLSLFLFLYFKWYMQN---GSFNWYLL
19 Mmoatsii_NCTC10158_V5E84_00290             -----------K----LLKWSKIFALICTS-PFWTFAHSWE-CCKQWFKSVLGNKMVGFTPTRSIDKGKKKVSNWSKIKYYVLTSFLTLLVLIGLN-FVMVYF----NL---LDWKWWEQ
20 Mmoatsii_NCTC10158_V5E84_00295             -----------K----LLKSSKTFELLCVV-PFWTFAHSWE-CCKQWFKSVLGNKMVGFTPTRSIDKGKKKVSNWSKIKYYVLTSFLTLLVLIGLN-FVMVYF----NL---LDWKWWEQ
21 Mmiroungigenitalium_ES2806-GEN_WP_171111006.1  IAIVS----KRK----W--LVPFQIIFYPVITFIMYTSLLVDCWIKAFITIFGIKAKFI-TTPKT---NEKISFGKKLLTNLIPISALLI-IASLVIVNFVFV---------HIISI---
22 Mzalophidermidis_CSL9942_WP_216505629.1    IAIVS----KRK----W--LVPFQIIFYPVITFIMYTSLLVDCWIKAFITIFGIKAKFV-TTPKT---NEKISFGKKILANIIPISTLLI-IATLLIVNFVFV---------HIISL---
23 Mequigenitalium_T37_UUD36799.1             ILNQC------K----Y--IGIRKTLIYLVLSFLLMFSIFTLSLRTIISIFFGKKSGFI-VTPKE---NKKLRFWAIIKYCFLDILVFLI-LVT---VSVLIS---------YYFA----
24 Mbovigenitalium_51080_ENY68771.1           LLIQH------K----Y--ISFPRFILFLIMSHFLIYSLFYINLKSMFLTIFGKKPTFV-VTPKN---NTKIDFKTFIKASWFEILLGFA-LISLIFVN---------------------
25 Mprimatum_ATCC25948_WP_029513203.1         ILNQH------K----Y--ISFFSTIFYFIISFTIIFSIFICSIKMILFSIFHKKPNFI-VTPKK---NYKFKLLMFIKQFWFEFLLCVL-MIVFCSLPMCIY---------GTDK----
26 Mfelifaucium_ATCC43428_WP_051590866.1      FIYWI------G----R--MNFFKFIIYFPLVFVLYGSMFLTSLISAILGIFGKKATFI-VTPKS---SYKYKFVDMFKYQWKELIFSLV-LIGIS-----------------LLN----
27 Mmucosicanis_1642_TQC51467.1               IIYWL------F----R--MNIFRFVLYFFSVFMLYGSMLTISLISAILGVFGKKAKFV-VTPKK---SYRYRFLDIFRFHWKEILFSLF-LLAIS-----------------LVN----
28 Mleachii_PG50_ADR24180.1                   FFVYA------K----S--KKIFFVIPFFIVSIINYTSLTPMMIRTVILGFFNKKAVFL-VTPKD---KNKIPLRYVILHSIEPLIFSFC-ISLLI-----------------YFS----
29 Mcapricolumcapricolum_14232_KEZ20687.1     FFVYA------K----S--KKIFFVIPFFIASIINYTSLTPMMIRTVILSLFNKKAVFL-VTPKD---KNKIPLRYVILHSIEPLIFSFC-VSLLI-----------------YFS----
30 Mcapricolumcapripneumoniae_M1601_AOQ21831.1  FFVYA------K----S--KKIFFVIPFFIASIINYTSLTPMMIRTVILSLFNKKAVFL-ITPKD---KNKIPLRYVILHSIEPLIFSFC-VSLLI-----------------YFS----
31 Magalactiae_14628_EIN15433.1               ALHLS------K----S--RNILLAIPVFLVTTIVYTAMNLSLVFAVINGLFNKKLKFI-VTPKE---SKKIPFKYMLLHSIIPFLFGAA-VLVAT-----------------YYS----
32 Mbovis_F11436_V5E85_03235                  AINLA------K----S--KNILLALPVFILTTIVYTAMNTSFVLSVFNGLLNKKIKFI-VTPKE---SVKIPFKYIIIHSIVPFLFGAA-TLVLT-----------------YFS----
33 Mmycoidescapri_PG3_EXU60752.1              AIHHS------K----N--KKIIWIIPNFIATIATYTSLTITLLLSVFLALFNRKPKFI-VTPKT---NTKISLKYIILHSLIPILFGAV-VILLT-----------------YFS----
34 Mmycoidescapri_PG3_EXU60168.1              VLELS------K----Q--RKLGWATPSFISTIITYTSMTIFLVASSFFALFNKRLKFV-VTPKE---SKKMKFAYVMAHSIVPIIFGIS-VAVLT-----------------YFS----
35 Mferiruminatoris_14822_WFQ92340.1          VLEFS------K----Q--RRLIWALPTFIGTIITYTSMTVFLVASSLLGLFNKRVKFV-VTPKE---SKKIKFIYIVLHSLIPIIFGVG-VAVLT-----------------YFA----
36 Mpirum_ATCC25960_WP_162150122.1            LIFMI-----QR----K--ENFLNLLGYLLFYLFFYPSLITFCIRIFTLSIFRKKAKFI-VTPKN---SKKYTIWSAIKLNIWDIFIYFL-FLSISIISVFLL---------GTFK----
37 Mpirum_ATCC25960_WP_052663068.1            IIFLI------R----K--ENFFHLLGYLFFCCLLYPSMIISSILTFTFSIFGKKAKFI-VTPKN---SKKYTFWEAIKFNKLELISSIV-LITLITLLTIFV---------NNFQ----
38 Miowae_695_EGZ31427.1                      FIYYL------G----K--VNFFKLIVILLFFYILYASLFVSVIISIFEALIGKKLNFV-VTPKKE--TKRISFIDAIKFNIIELIVGVI-LIIALIISFIYI---------PNIN----
39 Mpenetrans_HF-2_BAC44425.1                 TMFYW------K----K--VNFFHLVLVLLFQYLVYLSLFTSSITAVIKGLFGKRFSFF-VTPKKE--GEKVSIWYAIKTNIVEIIVSTI-LLTSVVLISIFI---------PKIG----
```

```
                                                       721         .         .         :         RxxxR     .         .       DxSxxG    8         .         .         .         . 840
 1 Mfermentans_PG18_BAH69904.1                         ------------------------------------------------------------------------------------------------------------------------
 2 Mbovigenitalium_51080_ENY68992.1                    ------------------------------------------------------------------------------------------------------------------------
 3 Mcaviae_G122_WP_126118392.1                         ------------------------------------------------------------------------------------------------------------------------
 4 Melephantis_ATCC51980_WP_027334204.1                ------------------------------------------------------------------------------------------------------------------------
 5 Malvi_ATCC29626_WP_052663867.1                      ------------------------------------------------------------------------------------------------------------------------
 6 Mferiruminatoris_14822_WFQ92977.1                   ------------------------------------------------------------------------------------------------------------------------
 7 Mmycoidescapri_95010_CBW54549.1                     ------------------------------------------------------------------------------------------------------------------------
 8 Mmycoidescapri_95010_CBW53832.1                     ------------------------------------------------------------------------------------------------------------------------
 9 Mmycoidesmycoides_PG1_CAE76760.1                    ------------------------------------------------------------------------------------------------------------------------
10 Malligatoris_A21JP2_EFF41433.1                      ------------------------------------------------------------------------------------------------------------------------
11 Mcrocodyli_MP145_ADE19752.1                         ------------------------------------------------------------------------------------------------------------------------
12 Mgallinarum_DSM19816_WP_027332911.1                 ------------------------------------------------------------------------------------------------------------------------
13 Mcalifornicum_HAZ106_1_WP_125075606.1               ------------------------------------------------------------------------------------------------------------------------
14 Mbovigenitalium_51080_WP_051043965.1                ------------------------------------------------------------------------------------------------------------------------
15 Csphaeroides_BcsA_WP_011338158.1                    WVAFPGDRSV-LLVVGGWAVLNVLLVGFALRAVAEKQQRRAAPRVQMEVPAEAQIPAFGNRSLTATVLDASTSGVRLLVRLPGVGDPHPALEAGGLIQFQPKFPDAPQLERMVRGRIRSA
16 Miowae_695_WP_004024575.1                           WLIIFFNLYFGVVWMGVFSYLVLWYINFIP--YNSN----FNRNSFIEC----------KEII---------------------------------------------------------
17 Mgirerdii_VCU_M1_AIV03393.1                         ILFWIAIYPLGFMWLSSIAYIILAITSKII--SNPN----YNDEDELKNIC--------LESM-----KFEKTINKFYLDH-------------------PEIKKSERLIKYE-------
18 Mmoatsii_NCTC10158_V5E84_00300                      FLFIALSSISGMFFISYFSTIILYLLSFIE--NNKD----YNDEKFVEY-N--------NDYV-----KFTNLKDKFFKNN-------------------LDIERFC-------------
19 Mmoatsii_NCTC10158_V5E84_00290                      FLIIFADVIILYVFLGNISTIILFLMSFIK--VNKS----YSPLDFVKF-D--------NDYI-----NFTTIKNDFFDKN-------------------KDIERFC-------------
20 Mmoatsii_NCTC10158_V5E84_00295                      FLIIFADVIILYVFLGNISTIILFLMSFIK--VNKS----YSPLDFVKF-D--------NDYI-----NFTAIKNDFFDKN-------------------KDIERFC-------------
21 Mmiroungigenitalium_ES2806-GEN_WP_171111006.1       ---YFIVYLTISFVIPIIFRILFSLLSNIE--MKTK------------------------------------------------------------------------------------
22 Mzalophidermidis_CSL9942_WP_216505629.1             ---YFIVYLTVTFVIPIIFRVLFSLLSNMK--IKIN------------------------------------------------------------------------------------
23 Mequigenitalium_T37_UUD36799.1                      ---WQSIFIFLPFIFGSISSIIFASFTNIN--TLTW----KSRE--RNFLN-F---------------ITCDTGGRKWKNM-------YSFQ----------------------------
24 Mbovigenitalium_51080_ENY68771.1                    ----KIFLFNIIFIIGLFSSFVLTILGNSN--NLKR----VSS---IKFEG---------------------FANNKFKNK-------FALN------KVVKI-----YKQRGK------
25 Mprimatum_ATCC25948_WP_029513203.1                  ---WHTLFFAFPFVWGFFGVLFLSIAGNNN--NSY-------------------------------------------LKI-------YESK------LLSKI-----FKEGPKGQVRS-
26 Mfelifaucium_ATCC43428_WP_051590866.1               ---FRNIWPVSLITISAILSMFLIYLSNIQ--YNQS----KIEIIDQNSSR-VSIQKIQKIFI-----KQCNYDL----KL-------YEQF------NLNKIQRTKINTQ---------
27 Mmucosicanis_1642_TQC51467.1                        ---FRNIWPVLLLVIPGICSIILIFFSNKR--YPHS----RTKTIDAKTAR-LSINKIQKDFL-----RYCDYDL----KL-------PESF------KQFYIQENCKTDKRSNNQINTN
28 Mleachii_PG50_ADR24180.1                            ---WGTVVPTLIITVPLALTPFIIVLSNFK--MNDK----EEKLSKTKRSF---------------------------------------------------------------------
29 Mcapricolumcapricolum_14232_KEZ20687.1              ---WGTVVPTLIITVPLALTPFIIVLANFK--MNDK----KEKLSKTKRSF---------------------------------------------------------------------
30 Mcapricolumcapripneumoniae_M1601_AOQ21831.1         ---WGTVVPTLIITVPLALTPFIIVLANFK--MNDK----KEKLSKTKRSF---------------------------------------------------------------------
31 Magalactiae_14628_EIN15433.1                        ---WGTVVPTLIVSIPCLLFPIVITFSNIP--LAKR----ATKQKQRARAG-AQQK----------------------------------------------------------------
32 Mbovis_F11436_V5E85_03235                           ---WGTVVPTLIVSIPCLLFPFAMTFSNIS--MTKR----VANKSKRQI-----------------------------------------------------------------------
33 Mmycoidescapri_PG3_EXU60752.1                       ---WDSVLPTLVISLPCLLTPFIILFSNFK--INAK------------------------------------------------------------------------------------
34 Mmycoidescapri_PG3_EXU60168.1                       ---WHTILPTLIVSLPCVLSPFLIASSNIN--LKEK----TVKKLKQNV-----------------------------------------------------------------------
35 Mferiruminatoris_14822_WFQ92340.1                   ---WHTILPTLIISLPCVLTPFLIATSNIR--LRTK----EKVKNKHKQKQ-NKMKTI--------------------------------------------------------------
36 Mpirum_ATCC25960_WP_162150122.1                     ---YELIFWYVFFTIPNIFIIFFTLMSNKK--NEKQ----NYQSYNL-------------------------------------------------------------------------
37 Mpirum_ATCC25960_WP_052663068.1                     ---YVLILWLVFFIIPFLSTPFLTLLSNKS--VVVK----N-------------------------------------------------------------------------------
38 Miowae_695_EGZ31427.1                               ---FFTYVWVIILIIPMFLTVPLTLMSNIK--LKNK----NHE-MENKYVI---------------------------------------------------------------------
39 Mpenetrans_HF-2_BAC44425.1                          ---LFGLLWVFLTICPLYMSIVFTMVPNIK--INNF----HRNNVTNQYSS-LVTFP---------------------------------------------------------------
```

```
                                                          841          :         .         .         .         .         9         .         .         .         .         :         . 960
 1 Mfermentans_PG18_BAH69904.1                                ------------------------------------------------------------------------------------------------------------------------
 2 Mbovigenitalium_51080_ENY68992.1                           ------------------------------------------------------------------------------------------------------------------------
 3 Mcaviae_G122_WP_126118392.1                                ------------------------------------------------------------------------------------------------------------------------
 4 Melephantis_ATCC51980_WP_027334204.1                       ------------------------------------------------------------------------------------------------------------------------
 5 Malvi_ATCC29626_WP_052663867.1                             ------------------------------------------------------------------------------------------------------------------------
 6 Mferiruminatoris_14822_WFQ92977.1                          ------------------------------------------------------------------------------------------------------------------------
 7 Mmycoidescapri_95010_CBW54549.1                            ------------------------------------------------------------------------------------------------------------------------
 8 Mmycoidescapri_95010_CBW53832.1                            ------------------------------------------------------------------------------------------------------------------------
 9 Mmycoidesmycoides_PG1_CAE76760.1                           ------------------------------------------------------------------------------------------------------------------------
10 Malligatoris_A21JP2_EFF41433.1                             ------------------------------------------------------------------------------------------------------------------------
11 Mcrocodyli_MP145_ADE19752.1                                ------------------------------------------------------------------------------------------------------------------------
12 Mgallinarum_DSM19816_WP_027332911.1                        ------------------------------------------------------------------------------------------------------------------------
13 Mcalifornicum_HAZ106_1_WP_125075606.1                      ------------------------------------------------------------------------------------------------------------------------
14 Mbovigenitalium_51080_WP_051043965.1                       ------------------------------------------------------------------------------------------------------------------------
15 Csphaeroides_BcsA_WP_011338158.1                           RREGGTVMVGVIFEAGQPIAVRETVAYLIFGESAHWRTMREATMRPIGLLHGMARILWMAAASLPKTARDFMDEPARRRRRHEEPKEKQAHLLAFGTDFSTEPDWAGELLDPTAQVSARP
16 Miowae_695_WP_004024575.1                                  ------------------------------------------------------------------------------------------------------------------------
17 Mgirerdii_VCU_M1_AIV03393.1                                ------------------------------------------------------------------------------------------------------------------------
18 Mmoatsii_NCTC10158_V5E84_00300                             ------------------------------------------------------------------------------------------------------------------------
19 Mmoatsii_NCTC10158_V5E84_00290                             ------------------------------------------------------------------------------------------------------------------------
20 Mmoatsii_NCTC10158_V5E84_00295                             ------------------------------------------------------------------------------------------------------------------------
21 Mmiroungigenitalium_ES2806-GEN_WP_171111006.1              ------------------------------------------------------------------------------------------------------------------------
22 Mzalophidermidis_CSL9942_WP_216505629.1                    ------------------------------------------------------------------------------------------------------------------------
23 Mequigenitalium_T37_UUD36799.1                             ------------------------------------------------------------------------------------------------------------------------
24 Mbovigenitalium_51080_ENY68771.1                           ------------------------------------------------------------------------------------------------------------------------
25 Mprimatum_ATCC25948_WP_029513203.1                         ------------------------------------------------------------------------------------------------------------------------
26 Mfelifaucium_ATCC43428_WP_051590866.1                      ------------------------------------------------------------------------------------------------------------------------
27 Mmucosicanis_1642_TQC51467.1                               KN----------------------------------------------------------------------------------------------------------------------
28 Mleachii_PG50_ADR24180.1                                   ------------------------------------------------------------------------------------------------------------------------
29 Mcapricolumcapricolum_14232_KEZ20687.1                     ------------------------------------------------------------------------------------------------------------------------
30 Mcapricolumcapripneumoniae_M1601_AOQ21831.1                ------------------------------------------------------------------------------------------------------------------------
31 Magalactiae_14628_EIN15433.1                               ------------------------------------------------------------------------------------------------------------------------
32 Mbovis_F11436_V5E85_03235                                  ------------------------------------------------------------------------------------------------------------------------
33 Mmycoidescapri_PG3_EXU60752.1                              ------------------------------------------------------------------------------------------------------------------------
34 Mmycoidescapri_PG3_EXU60168.1                              ------------------------------------------------------------------------------------------------------------------------
35 Mferiruminatoris_14822_WFQ92340.1                          ------------------------------------------------------------------------------------------------------------------------
36 Mpirum_ATCC25960_WP_162150122.1                            ------------------------------------------------------------------------------------------------------------------------
37 Mpirum_ATCC25960_WP_052663068.1                            ------------------------------------------------------------------------------------------------------------------------
38 Miowae_695_EGZ31427.1                                      ------------------------------------------------------------------------------------------------------------------------
39 Mpenetrans_HF-2_BAC44425.1                                 ------------------------------------------------------------------------------------------------------------------------
```

```
                                                    961        ] 968
 1 Mfermentans_PG18_BAH69904.1                          --------
 2 Mbovigenitalium_51080_ENY68992.1                     --------
 3 Mcaviae_G122_WP_126118392.1                          --------
 4 Melephantis_ATCC51980_WP_027334204.1                 --------
 5 Malvi_ATCC29626_WP_052663867.1                       --------
 6 Mferiruminatoris_14822_WFQ92977.1                    --------
 7 Mmycoidescapri_95010_CBW54549.1                      --------
 8 Mmycoidescapri_95010_CBW53832.1                      --------
 9 Mmycoidesmycoides_PG1_CAE76760.1                     --------
10 Malligatoris_A21JP2_EFF41433.1                       --------
11 Mcrocodyli_MP145_ADE19752.1                          --------
12 Mgallinarum_DSM19816_WP_027332911.1                  --------
13 Mcalifornicum_HAZ106_1_WP_125075606.1                --------
14 Mbovigenitalium_51080_WP_051043965.1                 --------
15 Csphaeroides_BcsA_WP_011338158.1                     NTVAWGSN
16 Miowae_695_WP_004024575.1                            --------
17 Mgirerdii_VCU_M1_AIV03393.1                          --------
18 Mmoatsii_NCTC10158_V5E84_00300                       --------
19 Mmoatsii_NCTC10158_V5E84_00290                       --------
20 Mmoatsii_NCTC10158_V5E84_00295                       --------
21 Mmiroungigenitalium_ES2806-GEN_WP_171111006.1        --------
22 Mzalophidermidis_CSL9942_WP_216505629.1              --------
23 Mequigenitalium_T37_UUD36799.1                       --------
24 Mbovigenitalium_51080_ENY68771.1                     --------
25 Mprimatum_ATCC25948_WP_029513203.1                   --------
26 Mfelifaucium_ATCC43428_WP_051590866.1                --------
27 Mmucosicanis_1642_TQC51467.1                         --------
28 Mleachii_PG50_ADR24180.1                             --------
29 Mcapricolumcapricolum_14232_KEZ20687.1               --------
30 Mcapricolumcapripneumoniae_M1601_AOQ21831.1          --------
31 Magalactiae_14628_EIN15433.1                         --------
32 Mbovis_F11436_V5E85_03235                            --------
33 Mmycoidescapri_PG3_EXU60752.1                        --------
34 Mmycoidescapri_PG3_EXU60168.1                        --------
35 Mferiruminatoris_14822_WFQ92340.1                    --------
36 Mpirum_ATCC25960_WP_162150122.1                      --------
37 Mpirum_ATCC25960_WP_052663068.1                      --------
38 Miowae_695_EGZ31427.1                                --------
39 Mpenetrans_HF-2_BAC44425.1                           --------
```
